# Multiscale Construction, Evaluation, and Application of Organoids

**DOI:** 10.1002/advs.202508534

**Published:** 2025-11-10

**Authors:** Wanting Ma, Zhenglin Dong, Zhu'anzhen Zheng, Long Bai, Xingcai Zhang, Jiacan Su

**Affiliations:** ^1^ MedEng‐X Institutes Shanghai University Shanghai 200444 China; ^2^ Organoid Research Center Institute of Translational Medicine Shanghai University Shanghai 200444 China; ^3^ National Center for Translational Medicine (Shanghai) SHU Branch Shanghai University Shanghai 200444 China; ^4^ Department of Orthopedics Xinhua Hospital Affiliated to shanghai jiao Tong University school of Medicine Shanghai 200092 China; ^5^ Wenzhou Institute of Shanghai University Wenzhou 325000 China; ^6^ World Tea Organization Cambridge MA 02139 USA

**Keywords:** organoids, multi‐scale construction, evaluation, application, artificial intelligence (AI)

## Abstract

Organoids serve as pivotal models in both basic and applied research, offering transformative potential in biomedical applications. Herein is presented a comprehensive multi‐scale perspective encompassing dual‐scale construction, four‐dimensional evaluation, triple‐point application, and an analysis of the current challenges faced by organoid technology, aiming to advance organoid research and its biomedical applications. Dual‐scale construction integrates micro‐scale and macro‐scale strategies to optimize material selection and spatial organization, thereby enhancing the biological fidelity of organoids. Four‐dimensional evaluation systematically assesses functional performance and long‐term stability at the molecular, cellular, organ, and in vivo levels, ensuring robust characterization. Triple‐point application explores the translational potential of organoids in basic research, preclinical studies, and clinical applications, with a focus on disease modeling, drug screening, and regenerative medicine. By refining construction methodologies, improving evaluation frameworks and facilitating clinical translation, this multi‐scale approach provides critical insights into optimizing organoid technology for biomedical research and therapeutic applications. The introduction of artificial intelligence (AI) empowers organoid research by enabling intelligent construction strategy screening, efficient multi‐scale image analysis, rapid multi‐omics data interpretation, and accurate preclinical assessment.

## Graphical abstract

Organoids are pivotal models with transformative biomedical potential. A comprehensive multi‐scale perspective is presented, encompassing dual‐scale construction, four‐dimensional evaluation, triple‐point application, and an analysis of the current challenges faced by organoid technology, aiming to advance organoid research and its biomedical applications. Furthermore, integrating artificial intelligence (AI) enables intelligent construction, efficient image and multi‐omics analysis, and precise preclinical assessment.

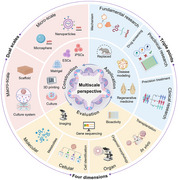

## 1 Introduction

Organoids are highly biomimetic miniature organ models formed by stem cells under three‐dimensional (3D) culture conditions, capable of simulating the structure and function of real organs in vitro.^[^ ^1^ ^]^ The emergence of organoid technology overcomes the limitations of traditional two‐dimensional cell culture and animal models in terms of tissue complexity and physiological relevance, making it widely applicable in both basic and applied research.^[^ ^2^ ^]^ In basic research, organoids provide a unique experimental platform for elucidating the molecular mechanisms of tissue development, cell differentiation, and organ function and can also be used to study biological processes such as tumorigenesis,^[^ ^3^ ^]^ immune responses,^[^ ^4^ ^]^ and infection mechanisms.^[^ ^5^ ^]^ In applied research, organoids have become important tools for drug screening,^[^ ^6^ ^]^ toxicological assessment,^[^ ^7^ ^]^ personalized medicine,^[^ ^8^ ^]^ and regenerative medicine.^[^ ^9^ ^]^

However, due to the complexity and interdisciplinary nature of the process from organoid construction to its evaluation and application,^[^ ^10^, ^11^ ^]^ a single‐scale perspective alone is inadequate to comprehensively address the demands of research and application. During the construction phase, the formation of organoids from single cells to 3D structures relies on the synergy between cells and the microenvironment.^[^ ^12^ ^]^ Current research is limited by a singular approach to construction, resulting in multiple challenges concerning construction complexity,^[^ ^13^ ^]^ standardization,^[^ ^14^ ^]^ scalability,^[^ ^15^ ^]^ and physiological relevance.^[^ ^16^, ^17^ ^]^ For example, the cellular composition and spatial structure of organoids often fail to fully replicate the complexity of real organs, resulting in insufficient reflection of specific organ functions.^[^ ^16^ ^]^ The difficulty in standardization leads to a lack of comparability between results from different laboratories, affecting research reproducibility. The industrial production of organoids is limited by the efficiency and repeatability of the culture system. In addition, the in vitro culture environment is difficult to achieve consistency with the in vivo environment, so the physiological relevance of organoids needs to be improved.^[^ ^18^ ^]^ The lack of unified standards for organoid evaluation indicators makes the results of different experimental studies incomparable, limiting the accuracy of organoids and the development of subsequent research. Dynamic and accurate detection of the formation and maturation of organoids is still a technology that needs to be developed at present. The ambiguity of organoids application areas further hinders the promotion of organoid technology. Currently, the scope of applicability and functional evaluation criteria for organoids in disease modeling, drug screening, and personalized medicine are still unclear. For example, in personalized medicine, how to validate clinical efficacy, define application boundaries, and establish standardized operational procedures remain urgent issues that need to be addressed. These problems limit the effectiveness of organoid functions and hinder their industrialization and practical application.

**Figure 1:**
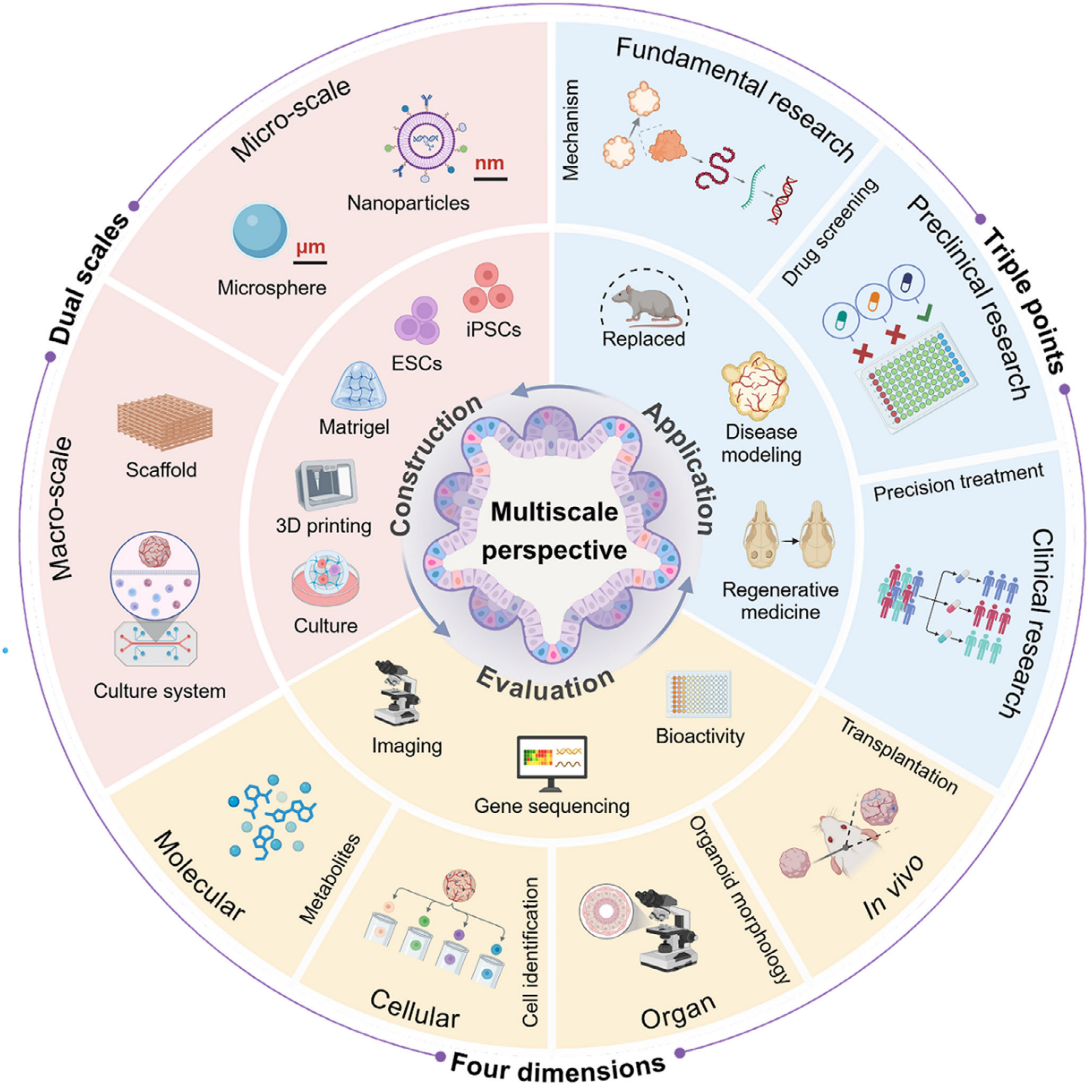
A multi‐scale perspective illustrating the strategies for constructing, evaluating, and applying organoids.

To this end, this article systematically reviews the development of organoid technology, and combines multidisciplinary knowledge from a multi‐scale perspective to elaborate on the strategies for constructing, evaluating, and applying organoids (**Figure** **1**). The aim is to assist scientists in optimizing the biological characteristics of organoids at different levels. Firstly, by integrating both micro and macro‐scale designs, we explore optimization pathways in material selection and spatial structure construction for organoids. Then, we comprehensively assess their functional performance and long‐term stability from four dimensions: molecular, cellular, organ and in vivo. Finally, from the perspectives of basic research, preclinical research, and clinical research, we conduct an in‐depth analysis of the practical applications of organoids in disease mechanism elucidation, drug screening, and regenerative medicine. We hope that the multi‐scale perspective presented in this article can provide important references for optimizing organoid functions, improving evaluation systems, and promoting their clinical applications, thereby opening new avenues for advancing their progress in scientific research and clinical applications.

## 2 The History of Organoid Development

**Figure 2:**
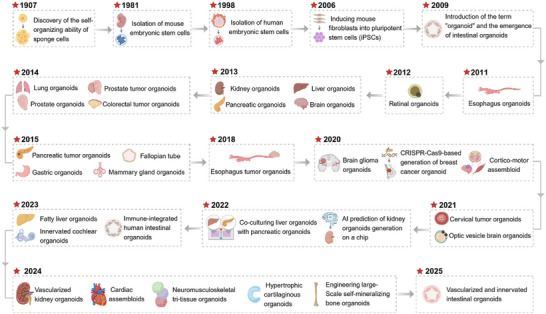
Historical timeline of the development of organoids.

The development of organoid technology has undergone a long and gradual process of exploration, with its scientific foundation stemming from in‐depth research on cellular self‐organization and microenvironmental regulation (**Figure** **2**). As early as 1907, scientists first discovered that sponge cells possess self‐organizing abilities,^[^ ^19^ ^]^ laying the theoretical foundation for subsequent studies. This was followed by the successful isolation of mouse embryonic stem cells (MEFs) in 1981^[^ ^20^ ^]^ and the establishment of human embryonic stem cells (hESCs) in 1998,^[^ ^21^ ^]^ marking significant milestones in the field of stem cells. The emergence of human‐induced pluripotent stem cells (iPSCs) have greatly promoted the development of organoids in 2006.^[^ ^22^ ^]^ At the same time, research on cell–cell interactions also provided significant guidance for organoid research. For instance, the cultivation of keratinocytes required the support from a 3T3 cell layer.^[^ ^23^ ^]^ Work by Li et al. revealed the key role of the extracellular matrix (ECM) in tissue‐specific gene regulation.^[^ ^24^ ^]^ Additionally, from the identification of collagen II from the ECM of chondrosarcoma cells to the introduction of the standard matrix gel Matrigel, a critical step was made in organoid culture.^[^ ^25^ ^]^

The term “organoid” was officially introduced in 2009 by the Dutch scientist Hans Clevers’ team. They used stem cells to induce a 3D intestinal organoid model, and opened organoids era.^[^ ^26^ ^]^ Subsequently, other soft tissue (normal or tumor) organoids were developed, including esophagus,^[^ ^27^ ^]^ retinal,^[^ ^28^ ^]^ kidney,^[^ ^29^ ^]^ liver,^[^ ^30^ ^]^ brain,^[^ ^31^ ^]^ pancreatic,^[^ ^32^ ^]^ lung,^[^ ^33^ ^]^ prostate,^[^ ^34^ ^]^ prostate tumor,^[^ ^35^ ^]^ colorectal tumor,^[^ ^36^ ^]^ pancreatic tumor,^[^ ^37^ ^]^ fallopian tube,^[^ ^38^ ^]^ stomach,^[^ ^39^ ^]^ mammary gland,^[^ ^40^ ^]^ esophagus tumor,^[^ ^41^ ^]^ and brain glioma.^[^ ^42^ ^]^ In 2020, the emergence of the cortico‐motor assembloid marked the realization of interactions between different organoids.^[^ ^43^ ^]^ Furthermore, the application of gene editing technologies,^[^ ^44^ ^]^ microfluidic technologies,^[^ ^45^ ^]^ and artificial intelligence^[^ ^46^, ^47^ ^]^ have greatly advanced the field of organoids. Researchers successfully developed breast cancer organoids with long‐term culture capability using CRISPR/Cas9 technology.^[^ ^48^ ^]^ The emergence of optic vesicle brain organoids in 2021 marked a breakthrough in organoid technology,^[^ ^49^ ^]^ while cervical tumor organoids enriched pathological modeling.^[^ ^50^ ^]^ By 2022, Microfluidics technology enabled co‐culture of different organoids on a chip.^[^ ^51^, ^52^ ^]^ At the same time, deep learning was able to predict the differentiation of iPSCs into kidney organoids.^[^ ^53^ ^]^ Subsequently, refinements in organoid technology enabled the development of fatty liver,^[^ ^54^ ^]^ innervated cochlear,^[^ ^55^ ^]^ immune‐integrated intestinal organoids,^[^ ^56^ ^]^ vascularized kidney organoids,^[^ ^57^ ^]^ cardiac assembloids,^[^ ^58^ ^]^ neuromusculoskeletal tri‐tissue organoids,^[^ ^59^ ^]^ and hypertrophic cartilaginous organoids.^[^ ^60^ ^]^ However, due to the mechanical environment requirements and the complexity of the mineralization process, the development of hard tissue bone organoids has been relatively slow. With advances in materials science and 3D culture technologies, large‐scale self‐mineralizing bone organoids constructed from hydroxyapatite‐based bio‐inks emerged in 2024.^[^ ^61^ ^]^ In 2025, vascularized and innervated intestinal organoids were successfully developed.^[^ ^62^ ^]^

In conclusion, organoid technology originated from early cell culture and stem cell research and, with continuous breakthroughs in key technologies, has gradually developed organoid models that highly simulate tissue structure and function, advancing toward the construction of complex organoid assemblies.

## 3 Multiscale Construction of Organoids

**Figure 3:**
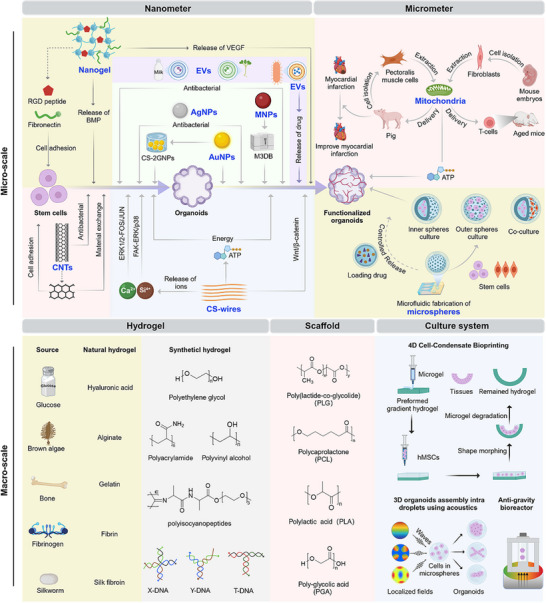
Multi‐scale construction of organoids, from microscopic to macroscopic.

Multi‐scale construction refers to the design and optimization of organoid construction across different scales, from microscopic to macroscopic (**Figure** **3**, **Table** **1**). At the micro‐scale, by regulating the cellular microenvironment, nano and micron materials are used to optimize cell behavior and promote tissue organization. At the macro‐scale, materials such as hydrogels and scaffolds are employed to provide mechanical support and create 3D structures, enhancing the stability and functional performance of organoids. In recent years, these multi‐scale construction strategies have played a unique role in organoid development, helping organoids transition from basic cell models to more complex tissue models.

**Table 1:** Comparison of biomaterials for organoid construction.

Scale	Type	Examples	Key features	Applications	Advantages	Reference
**Micro‐scale**	Nanomaterial	Nanogels	Porous architecture, high hydration capacity, functional modification	Airway organoid	Control of factor release, regulation of cell adhesion and differentiation	[74, 75, 76, 77, 78]
		Nanowires	Ion release, mechanical reinforcement, activation of signaling pathways	Neuro‐bone, gut, and liver organoids	Low cost, promotion of metabolism and function	[80, 81, 82, 83, 84, 85]
		Nanoparticles (AuNPs, AgNPs, MNPs)	Carrier of bioactive substances, antibacterial, magnetic response	Brain, liver, and salivary glands organoids	Precise regulation of stem cell differentiation, promotion of organoid maturation, reduction of infection risk	[86, 87, 88, 89, 90, 91, 92, 93, 94, 95, 96, 97, 98, 99, 100]
		Carbon nanotubes	Conductivity, high mechanical properties, antibacterial	Heart and intestinal organoids	Reduction of infection risk, promotion of organoid functionalization	[101, 102, 103, 104, 105, 106, 107, 108, 109]
		Extracellular vesicles	Lipid bilayer loaded with proteins and nucleic acids	Adipose organoid	Derived from various sources, regulation of microenvironment, signaling pathways, and functional molecule delivery	[110, 111, 112, 113, 114, 115, 116, 117, 118, 119, 120, 121, 122, 123]
	Micromaterial	Microspheres	ECM‐mimics, controlled release of factors	Osteo‐callus, heart and skin organoids	Morphology tunability, support of diverse culture strategies	[126, 127, 128, 129, 130, 131, 132, 133]
		Mitochondria	Provision of energy, regulation of metabolism	Intestinal organoid	Enhancement of cell activity and organoid maturation	[134, 135, 136, 137, 138, 139, 140, 141, 142, 143, 144, 145, 146]
**Macro‐scale**	Hydrogels	Natural hydrogel (HA, alginate, gelatin, fibrin, SF)	ECM‐mimics, high biocompatibility, abundant modifiable functional groups	Lung, brain, kidney, spinal cord, cortical, intestinal and tumor organoids	Derived from various sources, microenvironment, suitable for long‐term culture of diverse organoid types	[152, 153, 154, 155, 156, 157, 158, 159, 160, 161, 162, 163, 164, 165, 166, 167, 168, 169, 170, 171, 172, 173, 174, 175, 176, 177, 178]
		Synthetic hydrogel (PIC, PEG, PVA, PAAm and DNA hydrogels)	Controllable mechanics	Perineural, kidney, pancreatic and intestinal organoids	Tailorable with expandable functionality	[179, 180, 181, 182, 183, 184, 185, 186, 187, 188, 189, 190, 191, 192, 193, 194, 195, 196, 197, 198, 199, 200, 201, 202, 203, 204]
	Scaffolds	PGA, PLA, PLGA, PLG and PCL	Mechanical support	Cholangiocyte, brain and lung organoids	Construction of complex models for organoid culture	[205, 206, 207, 208]
	Culture systems	Microfluidics	Precise regulation of the microenvironment	Liver and islet organoids	High reproducibility, experimentation with various organoid co‐cultures	[51]
		4D bioprinting technique	Scaffold‐free, developmental mimicry	Cartilage organoid	Better recapitulation of development progression	[150]
		Anti‐gravity bioreactor, International Space Station	Simulated microgravity, low‐shear flow environment	3D cell spheroid, tumor‐myeloid organoids	Reduced culture duration and complexity	[149, 211]
		BAWs‐assisted droplet microfluidic	Rapid high‐throughput preparation in short term	Tumor organoid	High survival and functionality in long‐term organoid culture	[209]

### 3.1 Micro‐Scale

The micro‐scale assembly strategy of organoids refers to approaches that modulate the cellular microenvironment at the nanoscale to microscale, establishing a structural and biochemical foundation for their functional maturation and complexity. Nanomaterials provide highly precise 3D microenvironments that regulate cell attachment, proliferation, and differentiation, while also activating key signaling pathways to promote tissue maturation.^[^ ^63^, ^64^, ^65^, ^66^ ^]^ Micromaterials with controllable size, shape, and surface characteristics, optimize cell behavior and support the formation of organized tissues.^[^ ^67^, ^68^ ^]^

#### 3.1.1 Nanomaterial for Organoid Construction

Nanomaterials have been used in organoids technology for their unique physicochemical properties, high surface area and excellent regulation of ECM.^[^ ^69^, ^70^, ^71^, ^72^ ^]^ This section outlines the applications of nanogels, nanowires, carbon nanomaterials, metallic nanomaterials, magnetic nanomaterials, and exosomes in organoid construction.

##### Nanogels

Nanogels combine the properties of hydrogels and nanomaterials and are typically between 20 and 250 nanometers in size.^[^ ^73^ ^]^ Their porous structure and high hydration capacity provide a biocompatible microenvironment for cell adhesion, proliferation, and differentiation.^[^ ^74^ ^]^ For instance, cholesterol‐modified pullulan (CHPOA) nanogels, combined with RADA16 self‐assembling peptides, support the construction of complex tissue models.^[^ ^75^, ^76^, ^77^ ^]^ CHPOA nanogels enhanced cell adhesion and osteogenic differentiation through multi‐scale porous structures and functional modifications (e.g., fibronectin, RGDC peptides), mainly used in bone tissue regeneration.^[^ ^75^, ^77^ ^]^ In contrast, RADA16 self‐assembling peptides, with their highly ordered nanofiber structure, significantly promoted adipose stem cell differentiation and ECM secretion, while also supporting the formation of airway‐like structures (Airway‐Organoid) by human lung‐derived cells, expanding to more complex applications such as viral infection organoid models.^[^ ^76^ ^]^ Nanogels also exhibited advantages in delivering bioactive factors. For example, they could control the release of bone morphogenetic proteins (BMPs)^[^ ^77^ ^]^ or vascular endothelial growth factors (VEGF),^[^ ^78^ ^]^ which would promote vascularization and functional maturation in various organoid models. Furthermore, dynamic‐responsive nanogels could regulate the microenvironment according to cellular metabolic needs,^[^ ^79^ ^]^ providing more possibilities for organoid construction. Although nanogels offer significant advantages for organoid construction, their scalability and stability remain limited.

##### Nanowires

In recent years, nanowires have been used to culture complex organoids by regulating cell behavior and optimizing overall functionality. In neuro‐bone tissue model research, calcium silicate (CS) nanowires promoted the proliferation and differentiation of bone marrow mesenchymal stem cells (BMSCs) and Schwann cells (SCs) for specific tissue regeneration.^[^ ^80^, ^81^ ^]^ For example, CS nanowires enhanced the mechanical properties of hydrogels and promoted osteogenesis and neurogenesis, by releasing calcium and silicon ions (activating the ERK1/2‐FOS/JUN and FAK‐ERK/p38 signaling pathways, respectively).^[^ ^82^, ^83^ ^]^ Furthermore, CS nanowires also have been applied in intestinal and liver organoid cultures (GelMA‐2CS‐10M) as the vital component of hydrogels.^[^ ^84^ ^]^ Nanowires promoted stem cell proliferation and differentiation by releasing bioactive ions to activate the Wnt/β‐catenin pathway and then regulating mechanical properties and the nuclear localization of YAP proteins.^[^ ^85^ ^]^ This accelerated organoids maturation and functionalization, enhanced glucose uptake, and supported rapid growth by boosting ATP metabolism. Overall, CS nanowires, by synergistically enhancing both structural and functional outcomes, demonstrated immense potential as a low‐cost, safe, and efficient biomaterial in organoids culture.

##### Nanoparticles

Gold nanoparticles (AuNPs) exhibited excellent biocompatibility and could bind to the ECM or hydrogels, supporting cell adhesion, proliferation, and differentiation.^[^ ^86^ ^]^ By modifying functional molecules such as proteins and growth factors, they could mimic the in vivo microenvironment, promoting the maturation and functionalization of organoids. Additionally, AuNPs regulated the activity of cell membrane proteins and signaling pathways, enhancing cell differentiation and polarization while optimizing the interaction between organoids and the external physical microenvironment. This provided multi‐dimensional support for constructing organoids, such as brain and liver organoids.^[^ ^87^, ^88^ ^]^ AuNPs have been shown to be effective carriers for brain‐derived neurotrophic factor (BDNF).^[^ ^87^ ^]^ In iPSC‐derived brain organoids, AuNP‐BDNF could non‐toxically penetrate the organoid, significantly enhancing the expression of neurogenesis‐related genes (SOX2, MAP2) and promoting neurogenesis and maturation.^[^ ^87^ ^]^ A thermosensitive conductive hydrogel based on chitosan and gold nanoparticles (CS‐2GNPs, 1% GNPs) successfully mimicked the electromechanical properties of myocardial tissue, significantly enhancing the metabolism, proliferation, and expression of myocardial markers (Nkx‐2.5, α‐MHC) in mesenchymal stem cells (MSCs), thereby improving myocardial differentiation efficiency.^[^ ^89^ ^]^ Silver nanoparticles (AgNPs), due to their strong antibacterial properties and functional diversity, played a critical role in neural development regulation.^[^ ^90^, ^91^ ^]^ AgNPs could prevent contamination in organoid cultures, reduced the need for antibiotics, and optimized the metabolic microenvironment. However, AgNPs exhibited concentration‐dependent effects in brain organoid construction: at low concentrations, they inhibited apoptosis and promoted the proliferation of neural progenitor cells (NPCs), but they could interfere with cilia assembly, potentially leading to developmental abnormalities.^[^ ^92^ ^]^ At high concentrations, AgNPs significantly inhibited neurodifferentiation, increased apoptosis, weaken the formation of neurites and the establishment of neural circuits, and hindered organoids functional maturation.^[^ ^92^ ^]^ Therefore, careful control of AgNPs concentration is essential to avoid adverse effects.

Magnetic nanoparticles (MNPs) have shown significant value in the construction of organoids. MNPs could rapidly aggregate cells and precisely position them for enhancing cell–cell and cell–matrix interactions by magnetic adsorption.^[^ ^93^ ^]^ In organoid construction, MMPs could enhance the mechanical strength of the scaffold^[^ ^94^ ^]^ and as a delivery platform (growth factors, drugs or genes), for promoting the formation of vascularized and functionalized tissues.^[^ ^95^, ^96^ ^]^ For instance, MNPs were used to optimize ECM formation and then induce human tendon‐derived cells (hTDCs) to form tendon‐like tissues.^[^ ^97^ ^]^ The magnetic 3D bioassembly platform (M3DB) could utilize magnetic forces to orchestrate multicellular coordinated differentiation and construct salivary glands organoids with secretory functions.^[^ ^98^ ^]^ However, high‐dose internalization of MNPs excessive released of iron ions and caused the generation of reactive oxygen species (ROS) and oxidative stress.^[^ ^96^, ^99^ ^]^ Optimizing dosage, size, and surface modification is key to reducing toxicity. For example, chitosan coatings, lowering particle concentrations, or encapsulating iron oxide nanoparticles in calcium carbonate microcapsules through layer‐by‐layer self‐assembly techniques, have proved effective in mitigating their toxicity risks.^[^ ^97^, ^100^ ^]^

##### Carbon Nanotubes

Carbon nanotubes (CNTs) with high mechanical strength and excellent electrical conductivity,^[^ ^101^ ^]^ optimize the viscoelasticity of the matrix and promote cell adhesion, proliferation, and differentiation.^[^ ^102^ ^]^ CNTs improve the porosity of the matrix, enhancing nutrient transfer and waste removal, which facilitates metabolic activity in organoids.^[^ ^103^ ^]^ Moreover, studies have shown that CNTs exhibited antibacterial properties through physical perforation and the induction of oxidative stress, potentially reducing the risk of infections during organoid cultivation.^[^ ^102^, ^104^ ^]^ The application of CNTs has evolved from simple tissue models to complex organoids, and from supporting individual cell functions to fostering system‐level functional maturation. CNTs have contributed to angiogenesis and endothelial cell function,^[^ ^105^, ^106^ ^]^ regulated neural network function through signaling pathways,^[^ ^107^, ^108^ ^]^ enhanced cardiomyocyte differentiation and heart organoid construction,^[^ ^109^ ^]^ and expanded to optimizing the 3D growth environment, metabolic activity, and structural organization of intestinal organoids, highlighting their ability to support complex systems.^[^ ^103^ ^]^ Despite their advantages, carbon nanotube applications are still hindered by poor degradability and high functionalization complexity.

##### Extracellular Vesicles

Extracellular vesicles (EVs) are vesicles secreted by cells through multivesicular bodies (MVBs), characterized by a lipid bilayer structure.^[^ ^110^ ^]^ They have a wide range of origins, including animal,^[^ ^111^ ^]^ plant,^[^ ^112^ ^]^ and microbial sources.^[^ ^113^ ^]^ Among them, animal‐derived EVs can be classified based on their functions into ordinary EVs (for cell signaling), tumor EVs (carrying oncogenic molecules), and engineered EVs (for drug delivery and signal enhancement).^[^ ^114^ ^]^ EVs promote organoid differentiation and functional maturation by delivering functional molecules (such as proteins, nucleic acids, and lipids) and activating signaling pathways (e.g., PI3K‐Akt, VEGF, Notch).^[^ ^115^, ^116^, ^117^ ^]^ A typical example is EVs loaded with the CREG1 protein, which facilitated the formation of trophoblast‐derived organoids.^[^ ^116^ ^]^ EVs have found applications in cartilage repair, anti‐inflammatory processes, and immune modulation, providing new possibilities for organoid construction. For example, mesenchymal stem cell (MSC)‐derived EVs played an important role in maintaining matrix homeostasis and promoting tissue regeneration by regulating key signaling pathways (e.g., AKT, ERK, AMPK).^[^ ^118^ ^]^ This ability could be utilized to accelerate the formation and functional maturation of cartilage organoids. Bone marrow‐derived EVs enhanced chondrogenic activity, offering new tools for optimizing cellular functions in organoids. Milk‐derived EVs,^[^ ^119^ ^]^ when loaded with drugs, could provide robust environmental support for organoid models through their anti‐inflammatory properties and ability to protect the ECM. Additionally, plant‐derived EVs (P‐EVs) have antioxidant, anti‐inflammatory, and cell communication‐regulating effects.^[^ ^112^ ^]^ Combined with their biocompatibility and biodegradability, they could enhance the stability and physiological relevance of organoids. Bacterial‐derived EVs (B‐EVs) could regulate the microenvironment and immune responses, supporting the simulation of pathological conditions in organoid construction and enhancing organoid function as drug delivery carriers.^[^ ^113^ ^]^ Vascularized organoid construction is an important direction in organoid research, and EVs provide new strategies for this purpose. For example, salivary gland organoid EVs enhanced endothelial cell migration, proliferation, and angiogenesis, while adipose‐derived mesenchymal stem cell EVs optimized the cell microenvironment, improve immune status, and promote angiogenesis.^[^ ^120^ ^]^ A recent study demonstrated that adipose tissue‐derived extracellular vesicles promote vascularization of adipose organoids.^[^ ^121^ ^]^ These studies indicate that EVs from various sources regulate the microenvironment, signaling pathways, and functional molecule delivery, providing a solid theoretical foundation for future organoid applications. However, challenges remain in EV research, such as insufficient yield and purity, heterogeneity, lack of standards, storage deactivation, low engineering modification efficiency, unclear biological mechanisms, and ethical concerns.^[^ ^114^, ^122^, ^123^ ^]^ Technological innovation and standardization are needed to address these challenges and drive the development of EV‐based applications.

#### 3.1.2 Micromaterial for Organoid Construction

Micrometer‐scale materials, by precisely regulating their size, shape, and surface properties, provide a 3D microenvironment for organoid culture. Microsphere materials, by mimicking the physical properties of the ECM, support cell adhesion, proliferation, and differentiation, while their porous structure facilitates efficient exchange of nutrients and waste, creating an ideal metabolic environment.^[^ ^68^, ^124^ ^]^ Micrometer‐sized mitochondria, by providing energy support and regulating metabolic signals, significantly enhance cell vitality and functional maturation.^[^ ^125^ ^]^ This section focuses on the specific applications of microspheres in organoid culture and the key role of micrometer‐scale mitochondria in optimizing cell function.

##### Microsphere

Microsphere technology used in organoid culture mimics the ECM to provide an optimized 3D microenvironment that supports organoid maturation and functionalization. Through surface modification (such as peptides, proteins, or polysaccharides), microspheres enhance cell adhesion and enable controlled release of growth factors.^[^ ^68^, ^126^ ^]^ Their porous structure efficiently transports oxygen, nutrients, and waste, creating an ideal metabolic environment.^[^ ^68^ ^]^ To meet the diverse needs of organoid culture, microsphere materials encompass natural, synthetic, and composite materials, and their size, shape, and internal structure can be precisely controlled using techniques such as emulsification, spray drying, electrospinning, and microfluidics.^[^ ^127^ ^]^ These techniques support the application of inner spheres, outer spheres, and co‐culture systems, driving the development of novel microsphere materials and laying the foundation for organoid functionalization.

Inner spheres culture refers to encapsulating cells inside microspheres, where the microsphere material provides a 3D scaffold and supportive microenvironment, promoting the growth and differentiation of organoids. This method reduces the impact of external shear forces through physical barriers while enabling the encapsulation of growth factors inside the microsphere, allowing for continuous regulation of cell behavior and supporting the formation of organoid structures. GelMA‐based microsphere technology includes static microsphere construction (such as bone callus organoids) and dynamic bioprinting (such as ventricular organoids), extending the technology from basic 3D support to complex functionalized tissues. In 2022, GelMA microspheres were prepared using DLP 3D printing technology, successfully supporting the adhesion, differentiation, and osteo‐callus organoid formation of bone marrow mesenchymal stem cells (BMSCs), achieving in vivo bone defect repair.^[^ ^128^ ^]^ In 2023, GelMA microspheres prepared via microfluidics were used as bio‐ink to encapsulate human induced pluripotent stem cells (hiPSCs), printing a vascularized ventricular model.^[^ ^129^ ^]^ Cells proliferated and differentiated into beating functional cardiomyocytes under the protective barrier, showcasing a significant advancement in organoid fabrication.

Unlike inner sphere culture, outer sphere culture refers to the attachment of cells to the surface of microspheres, where they gradually form organoids through interactions with the microsphere matrix. This method is not limited by the size of the microspheres, allowing cells to expand freely, while enabling efficient nutrient exchange and signal molecule diffusion via direct contact with the culture medium. Moreover, outer sphere culture requires minimal surface modification and is highly compatible with various cell types. Researchers have combined poly (lactic‐co‐glycolic acid) (PLGA) microspheres with alginate methacrylate (AlgMA) matrix to create “groove‐ridge” microstructure.^[^ ^130^ ^]^ By adjusting the microsphere size (10–40 µm), they optimized the structure to promote the adhesion, proliferation, and osteogenic differentiation of bone marrow mesenchymal stem cells (BMSCs). This system significantly enhanced bone regeneration in a cranial defect model through M2 macrophage polarization and angiogenesis. Additionally, supramolecular microgels based on the supramolecular monomer aminopyridine ketone (UPy) further advance outer sphere culture technology by controlling the microgel size and mechanical properties through the adjustment of molar ratios.^[^ ^131^ ^]^ UPy‐cRGD peptide‐functionalized microgels facilitated the spontaneous assembly of normal human dermal fibroblasts (NHDFs) into microtissues within a day, demonstrating the flexible potential for cell culture.

Co‐culture methods integrate the advantages of both inner sphere and outer sphere cultures, enabling the construction of more complex and physiologically relevant organoid environments. By promoting interactions between different cell types, co‐culture systems better simulate tissue‐level organization and functionality, further advancing the field of organoid research. Researchers have used air microfluidics to fabricate alginate‐tyramine (ATA) soft microspheres (≈75 µm in radius).^[^ ^132^ ^]^ These microspheres are crosslinked through the tyramine groups under visible light, forming capillary‐like vascular networks. By adjusting the microsphere hardness and size, they optimized the vascular structure and density. When combined with hepatocytes and human umbilical vein endothelial cells (HUVECs), the HUVECs formed lumen‐like vessels within the gaps of the microspheres. The introduction of red blood cells simulated the circulatory environment, enabling the instant and uniform delivery of nutrients and oxygen, addressing the gradient diffusion issue. Similarly, a study fabricated microspherical skin organoids with a nucleus–shell configuration to recapitulate the bilayer structure of skin.^[^ ^133^ ^]^ The core, consisting of human dermal fibroblasts (HDFs) and collagen, mimics the dermis, whereas the outer shell is formed by human keratinocytes (HaCaT) adhering to the surface of the HDF spheroids to resemble the epidermis.^[^ ^133^ ^]^

Although inner sphere culture provides a 3D scaffold to support cell differentiation, its confined space may limit the size and functionality of organoids, and its efficiency in material transport and waste removal is relatively low. Additionally, scaling up production presents challenges in precise control. Outer sphere culture offers cells greater freedom and better nutrient access, but it relies on the properties of the microsphere surface, which may lead to cell detachment, and it lacks the 3D environmental simulation provided by inner sphere culture. Co‐culture systems integrate the advantages of both inner and outer spheres: inner spheres support cell differentiation, while outer spheres promote cell proliferation and nutrient exchange, thereby simulating more complex tissue environments, such as vascularized liver‐like tissues. However, this system requires careful balance of material properties, cell growth rates, and nutrient needs, and it increases the complexity of fabrication and monitoring. The challenges of spatial organization and stability management still need to be addressed.

##### Mitochondria

Mitochondria, known as the “powerhouses” of cells, provide energy through oxidative phosphorylation,^[^ ^125^ ^]^ and during stem cell differentiation, they shift from glycolysis to oxidative phosphorylation, accompanied by dynamic changes and reactive oxygen species (ROS) regulation.^[^ ^134^, ^135^, ^136^ ^]^ Moderate ROS levels promote differentiation, whereas excessive ROS can lead to oxidative stress and apoptosis.^[^ ^125^ ^]^ Mitochondria also influence cell communication and tissue development by regulating calcium signaling, the contact between the endoplasmic reticulum and mitochondria, and the release of metabolites.^[^ ^137^, ^138^, ^139^ ^]^ In organoid culture, mitochondria sense hypoxia and nutrient limitations, regulating autophagy and apoptosis to maintain cell survival.^[^ ^125^ ^]^ Metabolic reprogramming is crucial for achieving specific tissue functions.^[^ ^134^, ^140^ ^]^ However, direct mitochondrial delivery faces challenges such as poor stability, low efficiency, and potential induction of mitochondrial autophagy and immune responses from the recipient cells.^[^ ^141^ ^]^ Currently, there are two main types of mitochondrial delivery systems: natural extracellular vesicles for mitochondrial transfer and fusion‐based mitochondrial capsules (FMCs), which use electrostatic interactions between liposomes and the cell membrane to deliver mitochondria directly into the cytoplasm.^[^ ^142^ ^]^ There are very few studies on direct mitochondrial transplantation for organoid construction. However, research has demonstrated the feasibility of mitochondrial delivery in mammalian cells. For instance, in 2019, Shin et al. isolated mitochondria from pig thoracic muscle and injected them into the coronary artery.^[^ ^143^ ^]^ Their results showed that mitochondria were quickly absorbed by cardiomyocytes and significantly improved cardiac function, reducing infarction area in an ischemia‐reperfusion injury model. In 2023, Alian Zhang et al. extracted mitochondria from various cell types and verified the effectiveness of mitochondrial transplantation in mouse heart failure and dilated cardiomyopathy models.^[^ ^144^ ^]^ In 2024, Headley et al. transplanted mitochondria from young mouse embryonic fibroblasts (MEFs) into the T cells of aged mice, enhancing T cell metabolism and improving immune function.^[^ ^145^ ^]^ In 2025, a stem cell subpopulation enriched with “aged mitochondria” independently reconstructed the intestinal organoid microenvironment, demonstrating the powerful potential of organoid systems in modeling tissue self‐renewal and microenvironmental regeneration.^[^ ^146^ ^]^

### 3.2 Macro‐Scale

Macroscopic construction strategies focus on the overall stability and physiological function reproduction of tissues. From the perspective of macroscopic‐scale construction, organoid development primarily relies on the design and optimization of materials such as hydrogels and scaffolds. These materials provide essential 3D physical support and adjustable mechanical environments, forming the foundation for the long‐term cultivation and tissue organization of cell populations. Hydrogels, by tuning their elasticity, porosity, and biofunctionalization characteristics, support cell differentiation and functional maturation.^[^ ^147^ ^]^ Scaffold materials offer ideal mechanical support and material exchange conditions for cell adhesion and 3D growth.^[^ ^148^ ^]^ Furthermore, macroscopic‐scale culture systems (microfluidic technologies, 4D bioprinting, and acoustic levitation techniques), further accelerate the progress of organoid construction.^[^ ^45^, ^149^, ^150^ ^]^

#### 3.2.1 Hydrogel

Hydrogels, as three‐dimensional support materials for organoid culture, can be roughly divided into two categories: natural and synthetic.^[^ ^147^ ^]^ Natural hydrogels are derived from biomaterials and have abundant cell adhesion sites and bioactive groups. Synthetic hydrogels can be prepared by chemical synthesis according to needs and are highly adjustable.^[^ ^147^, ^151^ ^]^ This section aims to explore the applications and characteristics of natural and synthetic hydrogels in organoid construction.

##### Natural Hydrogel

Hyaluronic acid (HA), as a natural polysaccharide molecule, has excellent biocompatibility, high hydrophilicity and multiple biochemical functions, and is used in the construction of 3D cell culture systems. For example, HA‐PEGDT‐Matrigel hydrogels activated mechanotransduction signals by enhancing cell adhesion. iPSCs were expanded and differentiated into lung progenitor spheroids in 2D culture, followed by encapsulation in 3D hydrogels (HA‐PEGDT‐Matrigel hydrogels) to achieve differentiation and maturation of bronchial and alveolar epithelial cells for lung organoid culture.^[^ ^152^ ^]^ In brain organoids, HA‐Tyr‐modified hyaluronic acid (HA‐Tyr) and peptide amphiphiles (PAs) were used to create a dual‐component hydrogel that provided support comparable to commercial matrices like Matrigel, inducing organoid formation.^[^ ^153^ ^]^ In brief, after hiPSCs are seeded onto culture plates to induce the formation of embryoid bodies (EBs), the EBs are subsequently embedded in the hydrogel, followed by the addition of hydrogen peroxide to activate the HRP‐mediated oxidative coupling of HA‐Tyr, thereby promoting organoid formation. Liver cancer organoids can be encapsulated in HAMA‐SA dual‐network hydrogels, composed of methacrylated hyaluronic acid (HAMA) and sodium alginate (SA), which allow precise regulation of pore structure, enhance mechanical stability and mass transport efficiency, and thereby recreate the liver cancer microenvironment.^[^ ^154^ ^]^ Research indicates that hydrogels, through material diversity, structural optimization, and functional design, can precisely simulate the mechanical and biological microenvironment of specific tissues, supporting the differentiation, maturation, and functionalization of organoids. From healthy tissues to pathological models, the broad adaptability of hydrogels demonstrates their immense potential in organoid construction, disease research, and regenerative medicine.

Alginate is a natural polysaccharide derived from brown algae, known for its high biocompatibility, biological stability, chemical inertness, low toxicity, and low cost.^[^ ^155^ ^]^ Due to these properties, it is widely used in the biomedical field. Its unique ability to form gels can be achieved through divalent ion crosslinking, making it suitable for various tissue engineering and cell culture systems.^[^ ^155^ ^]^ With further chemical modification and process optimization, alginate now capable of encapsulating cells within its cross‐linked network, has demonstrated even more diverse application potential by providing a three‐dimensional microenvironment that supports organoid formation. For example, thiol‐ene crosslinked alginate hydrogels (NB‐Alg), created through chemical modification, can significantly reduce the fibrotic tendencies of kidney organoids, supporting their long‐term vitality and providing a new approach for the clinical translation of hiPSC‐derived kidney organoids.^[^ ^156^ ^]^ Beyond kidney organoid culture, alginate also shows excellent performance in other organoids and disease modeling. In one study, 1% alginate gel with moderate stiffness (around 1000 Pa) supported the generation of spinal cord organoids and neuronal maturation, while also replicating pathological features of neurodegenerative diseases like ALS, providing an important tool for disease modeling.^[^ ^157^ ^]^ Moreover, the application range of alginate has been further expanded through innovative processing techniques. High porosity alginate cryogels, prepared using low‐temperature freezing and lyophilization, successfully simulate tumor–immune interactions in the breast cancer microenvironment by regulating mechanical properties, which promoted macrophage polarization and the formation of breast cancer spheroids.^[^ ^158^ ^]^ These studies demonstrate that alginate, through mechanical property regulation, chemical modification, and process optimization, supports a wide range of biomedical applications from organ generation to disease modeling and cancer research.

Gelatin is a natural polypeptide material extracted from collagen, primarily derived from animal skin, bones, or connective tissues.^[^ ^159^ ^]^ It retains the cell adhesion motif Arg‐Gly‐Asp of collagen and has biodegradability.^[^ ^160^ ^]^ Gelatin contains a rich array of functional groups, making it easy to chemically modify and enabling efficient cell encapsulation. For instance, methacrylated gelatin (GelMA) possesses photopolymerizable properties and controllable mechanical performance, showing excellent performance in organoid culture. By adjusting the concentration and degree of substitution (DS) of GelMA, along with functional modifications (such as N‐cadherin peptides,^[^ ^161^ ^]^ hyaluronic acid,^[^ ^162^ ^]^ dextran and chondroitin sulfate^[^ ^163^ ^]^), the hydrogel can precisely regulate its mechanical properties and biological functions to meet the needs of different organoids.^[^ ^164^ ^]^ For example, GelMA‐Cad hydrogels enhanced neural differentiation for the generation of cortical organoids^[^ ^161^ ^]^; GelMA‐HA‐RGD hydrogels promoted the expansion and differentiation of cochlear progenitor cells through mechanical stiffness modulation;^[^ ^162^ ^]^ DexNB‐GelSH and CSNB‐GelSH hybrid hydrogels promoted the formation of liver organoids.^[^ ^163^ ^]^ By optimizing the mechanical properties and biocompatibility of gelatin‐based hydrogels, the physical and chemical microenvironments of specific tissues can be effectively simulated. This advancement has facilitated the diverse applications of organoids, ranging from endometrial and neural organoids to cochlear and liver organoids.^[^ ^161^, ^162^, ^163^, ^164^ ^]^ This demonstrates the key role and technical progression of hydrogels in supporting the differentiation, maturation, and functionalization of organoids.

Fibrin is a natural protein polymer formed from fibrinogen under the action of thrombin.^[^ ^165^, ^166^ ^]^ Due to its excellent biocompatibility, suitable mechanical properties, biodegradability, and abundant bioactive sites,^[^ ^167^ ^]^ fibrin has become an important matrix material in organoid culture. Its large porous structure not only facilitates cell migration and material exchange but also allows for the regulation of the degradation rate through cross‐linking density and functional modifications,^[^ ^168^ ^]^ supporting the long‐term stable culture of organoids embedded within the gel. Fibrin‐based hydrogels, such as those developed by Broguiere et al., combined with laminin, provide cell adhesion and differentiation signals, supporting the long‐term stable culture of epithelial organoids derived from both mouse and human sources (e.g., small intestine, liver, pancreas).^[^ ^169^ ^]^ In another study, Wen et al. incorporated protease inhibitors and 15% Matrigel into fibrin, combined with collagen fiber bundles, to promote the growth of intestinal organoids and the formation of endothelial cell vascular networks.^[^ ^170^ ^]^ These studies, through functionalization strategies (such as laminin, Matrigel, and collagen fiber bundles), have optimized the biological characteristics of fibrin, enhancing the growth environment for organoids. Furthermore, by promoting vascularization, they increase the complexity and functionality of organoids, demonstrating the broad application potential of fibrin hydrogels in the construction of various organoids.

Silk fibroin (SF) is a structural protein extracted from natural silk materials, such as silkworm silk or spider silk, composed of two heavy chains and two light chains connected by disulfide bonds.^[^ ^171^ ^]^ Its β‐sheet structure imparts high strength and elasticity to silk fibroin.^[^ ^171^ ^]^ Due to its tunable mechanical properties, biocompatibility, thermal stability, and high processability, SF has shown great potential in areas like bone tissue engineering and organoid 3D printing.^[^ ^172^ ^]^ SF hydrogels, through various crosslinking strategies and functional designs, support cell differentiation, tissue regeneration, and tumor simulation. For example, Calabrese et al. combined SF with poly‐L‐lysine (PL) or poly‐L‐glutamic acid (PG) to prepare SF‐PL and SF‐PG hydrogels, which promoted osteogenic differentiation or supported both osteogenesis and adipogenesis under non‐inductive conditions.^[^ ^173^ ^]^ Incorporating nanomaterials, such as nHA (nano‐hydroxyapatite) and GO (graphene oxide), enhanced the osteogenic capability of SF hydrogels (Bm@nHG‐SFD),^[^ ^174^ ^]^ while crosslinking SF with tannic acid (SF‐TA) imparted antioxidant and anti‐inflammatory properties, making it useful for bone‐cartilage repair.^[^ ^175^ ^]^ Further, the introduction of copper nanoparticles (CuTA@SF) activated the Nrf2 signaling pathway, further enhancing osteogenesis and cartilage formation.^[^ ^176^ ^]^ Furthermore, silk fibroin is increasingly used to encapsulate stem cells for organoid formation, offering a stable and supportive matrix that facilitates long‐term culture of organoids within hydrogels. By employing a double‐network design, DNA‐SF hydrogels regulate Wnt and TGF‐β pathways, promoting cartilage differentiation and organoid formation, facilitating joint cartilage regeneration and tissue fusion.^[^ ^177^ ^]^ Moreover, SF hydrogels combined with 3D‐embedded bioprinting technology have successfully supported low‐concentration collagen‐based bioinks to construct breast cancer models, simulating the complex microenvironment of tumor core regions and surrounding stroma, showcasing excellent printing precision and tumor characteristic reproduction capabilities.^[^ ^178^ ^]^ These studies, spanning osteogenesis, cartilage repair, and tumor modeling, highlight the adaptability and functional potential of SF hydrogels in various fields.

##### Synthetic Hydrogel

Polyisocyanopeptides (PIC) are thermoreversible hydrogels that are low‐viscosity solutions at low temperatures and gel upon heating (>18 °C) without the need for enzyme or light‐induced crosslinking.^[^ ^179^ ^]^ As shear‐thinning materials, the hardness and elasticity of PIC hydrogels can be precisely adjusted by controlling polymer concentration and crosslinking density, making them suitable for various hydrogel‐encapsulated organoid culture needs.^[^ ^180^ ^]^ Their thermosensitivity and tunable mechanical properties make them highly effective in supporting organoid growth and functionalization, with notable advantages over Matrigel. Ye et al. developed PIC hydrogels that promote the differentiation of adult liver stem cells into organoids with essential liver functions, achieving stable cultures for up to 14 passages.^[^ ^181^ ^]^ A 2024 study further confirmed that PIC hydrogels support the maturation of intrahepatic cholangiocyte organoids (ICOs) into cholangiocytes with apical‐basal polarity consistent with in vivo bile ducts.^[^ ^182^ ^]^ Moreover, PIC hydrogels functionalized with RGD peptides mimic the extracellular matrix (ECM) environment, supporting the differentiation of mouse mammary epithelial cells into bilayered mammary organoids with lumens.^[^ ^183^ ^]^ The stiffness of the matrix can be adjusted by changing the molecular weight and concentration of PIC. In addition, PIC‐RGD hydrogel was dissolved after cooling, which increased the cell recovery rate.^[^ ^183^ ^]^ In summary, these studies show that the adjustable mechanical properties of PIC hydrogels support the generation of various organoids, and low‐temperature solubility provides great convenience for cell recovery.

Polyethylene glycol (PEG) is a hydrophilic polymer with low immunogenicity.^[^ ^184^ ^]^ It can simulate ECM to provide a suitable environment for cell growth and differentiation. The mechanical properties of PEG can be adjusted by changing its molecular weight, the number and type of functional groups.^[^ ^184^ ^]^ PEG‐based hydrogel‐encapsulated stem cells were employed to facilitate and sustain three‐dimensional organoid formation. For example, in 2018, PEG‐4MAL hydrogels formed by covalent bonding of maleimide groups to cysteine peptides successfully supported the differentiation of hESCs and human induced hiPSCs into intestinal, lung, kidney, and brain organoids.^[^ ^185^ ^]^ Subsequently, the properties of PEG hydrogels were further optimized. In 2019, gelPEG hydrogels, combining the characteristics of PEG and gelatin, were crosslinked enzymatically.^[^ ^186^ ^]^ These hydrogels not only promoted the growth of liver organoids and early bone matrix mineralization but also formed capillary‐like networks comparable to Matrigel. In 2022, Chrisnandy et al. designed a crosslinked hybrid PEG hydrogel using both physical and chemical crosslinking strategies, which supported the long‐term culture of mouse intestinal organoids and exhibited a multicellular pattern similar to Matrigel.^[^ ^187^ ^]^ Additionally, the functionalization of PEG hydrogels further expanded their application range. In 2023, researchers covalently modified PEG molecules with collagen‐derived GFOGER peptides and fibronectin‐derived PHSRN‐K‐RGD peptides.^[^ ^188^ ^]^ Supporting embryonic stem cells (ESCs) differentiation into epithelial organoids (EEOs) by adjusting PEG stiffness(300 Pa).^[^ ^188^ ^]^ Cells attached to the surface of PEG hydrogels can also give rise to organoid formation. For example, in 2024, novel PEG‐based hydrogels, which were formed via photocrosslinking reaction of PEG‐norbornene with matrix metalloproteinase (MMP)‐degradable peptides and cell adhesion peptides, efficiently supported the construction of perineural organoids (PNOs).^[^ ^189^ ^]^

Polyvinyl alcohol (PVA) is an environmentally friendly material with biodegradability, adhesion, and mechanical strength suitable for cell survival.^[^ ^190^ ^]^ It can be cross‐linked into a three‐dimensional network hydrogel structure by physical, chemical, or radiation cross‐linking methods.^[^ ^191^ ^]^ For example, PVA‐SA hydrogels effectively supported the proliferation and differentiation of neural progenitor cells (NPCs) by improving the pore structure and degradation rate of the hydrogels and enhancing the response of related chemical signals (such as VAP).^[^ ^192^ ^]^ The functional modification of PVA by introducing thiol groups and RGD tripeptide, modulated its elastic modulus and supported the culture of pancreatic organoids embedded within the hydrogel.^[^ ^193^ ^]^

The tunable stiffness, biological inertness, high water absorption, mechanical and structural stability, and high permeability of polyacrylamide (PAAm) make it an ideal hydrogel material.^[^ ^194^ ^]^ By adjusting the concentration of crosslinking agents and the monomer ratio, PAAm hydrogels could provide various mechanical environments that influence cell differentiation and organoids formation. A study found that on the surface of soft 1 kPa hydrogels, human pluripotent stem cells (hPSCs) tended to differentiate into kidney progenitor cells and promote kidney organoids formation.^[^ ^195^ ^]^ These organoids successfully vascularized and integrated with endothelial cells after transplantation into chick embryos. Further studies revealed that functionalized PAAm hydrogels provided a controllable mechanical environment, supporting the formation of intestinal organoids on their surface.^[^ ^196^ ^]^ A stiffer matrix inhibited crypt folding, while a softer matrix promoted mechanical signaling that drove crypt formation and cell migration. These studies, spanning from cardiovascular to kidney and intestinal organoids, gradually unveil the critical role of matrix stiffness in organoid generation and cell behavior, providing a crucial tool for regulating the mechanical microenvironment.

DNA hydrogels, based on their multifunctionality (thermoreversibility, high mechanical stability, and excellent cell compatibility) and tunability, combined with innovative designs incorporating various materials, provide strong technical support for cell culture, tissue engineering, and regenerative medicine.^[^ ^197^ ^]^ DNA hydrogels form 3D networks through electrostatic interactions, hydrogen bonding and DNA hybridization.^[^ ^198^ ^]^ Their thermoreversible properties allow them to remain stable in various biological environments, while dynamic crosslinking enables precise regulation of cell behavior. For example, DNA hydrogels, when combined with polyacrylamide (PAAm) through in situ polymerization, form a dual‐network hydrogel that exhibited excellent mechanical properties and thermal stability (remaining gelled from 10 to 70 °C), while also supporting high cell viability (>90%) within the hydrogel matrix, making it suitable for 3D cell culture and imaging.^[^ ^199^ ^]^ Dynamic DNA hydrogels (DyNAtrix) supported organoid generation from MSCs and hiPSCs, enabling homogeneous embedding of cells within the matrix and achieving performance comparable to or even superior to Matrigel.^[^ ^200^ ^]^ The dynamic crosslinking nature of DyNAtrix hydrogels not only supported the proliferation and differentiation of various cells but also provided a tool for studying cell behavior in dynamic environments. When combined with 3D printing technology, supramolecular peptide‐DNA hydrogels enabled fast gelation, high mechanical strength, and the construction of complex tissue models, maintaining high cell survival rates and functionality.^[^ ^201^ ^]^ These hydrogels allow for precise structural design via DNA hybridization, and their unique hybridization properties provide dynamic controllability during cell encapsulation and tissue construction, effectively preventing cell damage. In promoting vascularization and bone regeneration, black phosphorus nanosheets (BPNSs)^[^ ^202^ ^]^ combined with DNA hydrogels, loaded with VEGF, significantly enhanced angiogenesis and osteogenic differentiation.^[^ ^203^ ^]^ Additionally, DNA hydrogels combined with amyloid fibrils (AF) and clay nanosheets form dynamic composite materials, providing fibrous networks and the release of bioactive ions (Si⁴⁺ and Mg^2^⁺), further enhancing the migration and functionality of HUVECs and BMSCs.^[^ ^204^ ^]^ The precise control of VEGF release by BPNSs and the mechanical reinforcement by clay nanosheets greatly improved the performance of these hydrogels in regenerative medicine. Compared to other hydrogels (such as Matrigel and PEG), DNA hydrogels have significant advantages in mechanical properties, biocompatibility, and multifunctional design, especially in constructing dynamic microenvironments, showcasing unique potential. In the future, combining artificial intelligence to design DNA hydrogel formulations and further optimizing their performance in cell regulation and tissue regeneration may drive significant advancements in this field.

#### 3.2.2 Scaffold

Synthetic biological scaffolds are support systems fabricated by precisely controlling the composition and microstructure of materials, featuring adjustable mechanical properties, porosity, and biocompatibility, which can mimic the physicochemical characteristics of the natural ECM.^[^ ^148^ ^]^ In organoid culture, synthetic scaffolds provide a 3D supportive environment for cells, promoting differentiation, proliferation, and spatial organization, and enhancing cell–material interactions by functionalizing growth factors or signaling molecules, thereby improving the functionality and stability of organoids.^[^ ^148^ ^]^ Polyglycolic acid (PGA) scaffolds supported the formation of continuous bile duct structures in cholangiocyte organoids (COs) and could successfully repair bile duct defects.^[^ ^205^ ^]^ Furthermore, functionalized polylactic acid (PLA) scaffolds induced adipose‐derived stem cells (ASCs) to differentiate into cartilage microtissues with glycosaminoglycan and type II collagen deposition.^[^ ^206^ ^]^ Poly(lactide‐co‐glycolide) (PLGA) microfibrous scaffolds could optimize the structure and function of brain organoids and promote the expression of forebrain‐specific markers by activating Wnt signal.^[^ ^207^ ^]^ The diameter and arrangement of PLGA microfibers significantly influence embryoid body (EB) development, enhancing forebrain region specificity and supporting higher levels of structural complexity. When combined with poly(lactide‐co‐glycolide) (PLG) and polycaprolactone (PCL) scaffolds, these materials facilitated the formation of airway‐like structures in lung organoids (HLOs).^[^ ^208^ ^]^ Although current scaffolds already possess good mechanical properties, their biodegradability and immunological compatibility still need to be optimized for future applications.

#### 3.2.3 Culture System

Organoid culture systems are technologies that simulate the 3D microenvironments of in vivo tissues to culture cells, enabling the construction of complex cell structures in vitro that replicate biological characteristics of native tissues. These systems precisely support cell‐to‐cell and cell‐to‐matrix interactions, promoting cell functionalization and differentiation. In recent years, the development of organoid culture systems has been driven by advancements in new materials and technologies. For instance, highly biomimetic hydrogel materials can provide 3D matrices to support cell growth, microfluidic chip technology allows precise control of the culture environment, and non‐contact technologies such as acoustics and bioprinting further enhance the controllability and diversity of the culture process.^[^ ^51^, ^149^, ^150^, ^209^, ^210^ ^]^ In 2022, a 4D bioprinting technique was used to construct a dual‐layer culture system, consisting of a gradient‐crosslinked hydrogel layer and a degradable microgel layer that supports cell aggregation.^[^ ^150^ ^]^ This system enabled the programmable shape transformation and dynamic release of human mesenchymal stem cell (hMSC) aggregates. By adjusting the deformation amplitude and degradation rate, the system optimized the intercellular interaction environment and successfully constructed “C” shaped and spiral‐shaped cartilage organoids. By incorporating the temporal dimension to simulate tissue development, this study offers new insights into optimizing culture systems and enables scaffold‐free organoid generation. By 2023, the introduction of acoustic levitation technology further simplified the organoid culture process, particularly showing significant advantages in the construction of 3D spheroids.^[^ ^149^ ^]^ The anti‐gravity bioreactor forms an acoustic standing wave pressure field, which allows hMSCs to rapidly aggregate into uniform and dense spheroids within 12 h. Compared to traditional methods, this approach not only shortens culture time but also reduces experimental complexity. Moreover, the system significantly increased the secretion levels of paracrine factors in the spheroids, particularly vascular endothelial growth factors (VEGF, IGF‐1, ANGPT2), while decreasing the expression of aging factors (such as p16 and p21), thus providing strong support for tissue regeneration and cell therapy. Furthermore, the International Space Station's microgravity environment facilitates the generation of advanced tumor‐myeloid organoids that model glioblastoma (GBM)–immune interactions with high fidelity, offering a powerful platform to study the immunosuppressive tumor microenvironment and screen future therapies.^[^ ^211^ ^]^ In 2024, research combined acoustic technology with droplet microfluidics to control the sound field generated by bulk acoustic waves (BAWs), enabling high‐throughput preparation of tumor organoid.^[^ ^209^ ^]^ By regulating the sound field formed by BAWs, the technology achieved rapid cell aggregation in droplets and the construction of diverse structures (e.g., spherical, linear, and cross‐linked). Experiments showed that this method could complete the assembly of thousands of droplets in just 1 min, significantly improving the tumor spheroid formation rate and uniformity (about 70% compared to 30% using traditional passive assembly methods). Additionally, the generated tumor spheroids exhibited high survival rates and biological functionality during long‐term culture, providing an efficient technological platform for drug screening, disease modeling, and personalized treatment.

Overall, this series of studies demonstrates the great potential of organoid culture systems in simulating tissue morphology, enhancing culture efficiency, and driving high‐throughput applications.

## 4 Evaluation Criteria of Organoids

**Figure 4:**
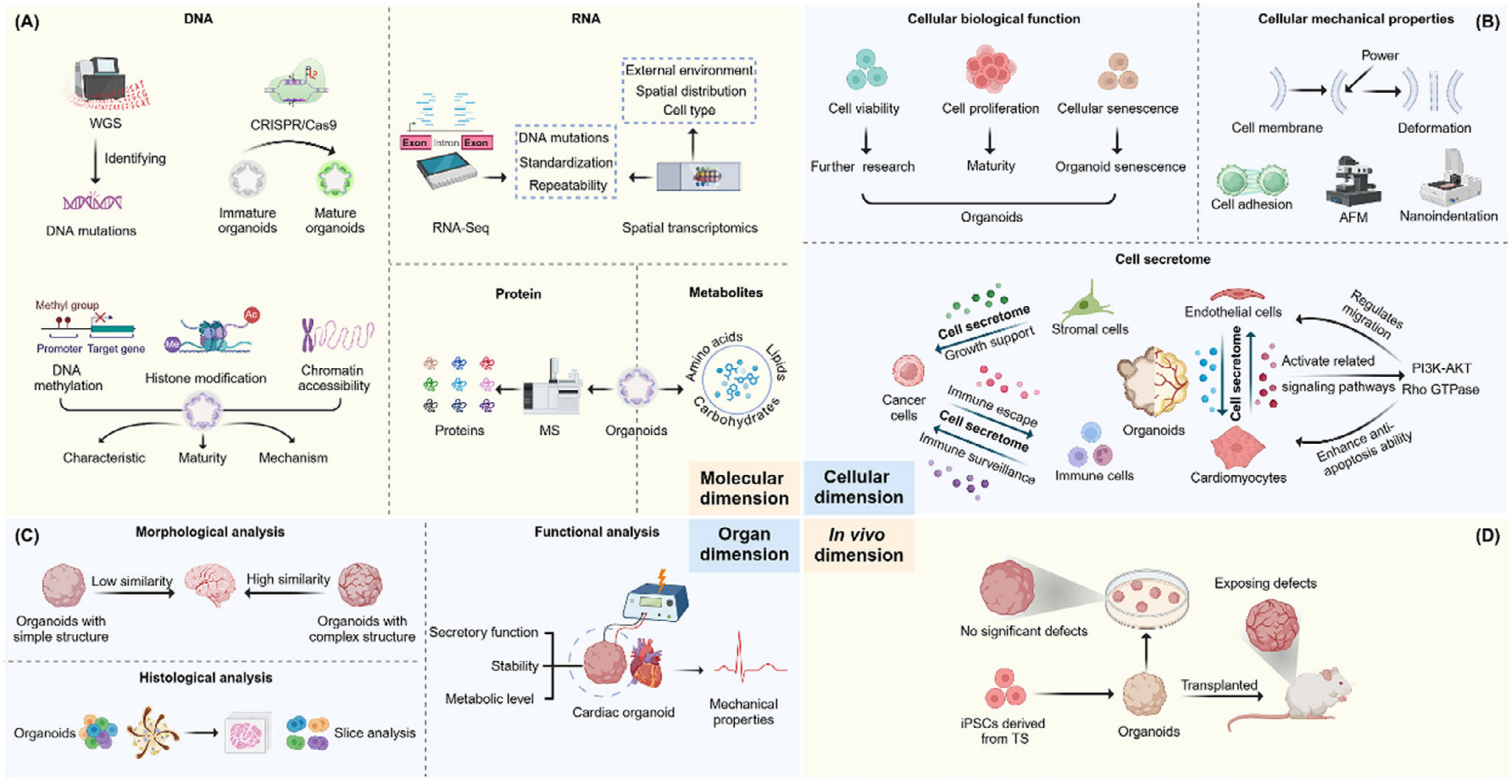
Evaluation criteria of organoids across four dimensions: molecular, cellular, organ and in vivo levels.

Establishing comprehensive evaluation criteria to assess the functionality and physiological similarity of organoids from multiple dimensions is crucial for the translational application of organoids. This section evaluates organoids from four dimensions (molecular, cellular, organ, and in vivo) (**Figure** **4**). Covers the molecular composition, cell composition, morphological structure, mechanical properties, etc. of organoids.

### 4.1 Molecular Dimension

Molecular‐level evaluation (genomics, transcriptomics, proteomics, and metabolites), could determine their similarity between organoids and target tissues. The discussion includes the stability of genetic information in DNA, gene expression profile in RNA, functionality of proteins and physiological activity of metabolites.

#### 4.1.1 DNA

Genomics research focuses on the arrangement, expression, and interactions of genes, as well as their regulation of biological functions.^[^ ^212^ ^]^ Whole Genome Sequencing (WGS) (Figure 4A) is a genomic technology that provides a comprehensive, high‐resolution map of the genome.^[^ ^213^ ^]^ WGS is particularly helpful in assessing whether organoids simulate genomic features of genetic diseases and cancers. For example, the Shi team conducted WGS on organoids derived from 84 pancreatic cancer patients and found that the organoids retained unique genomic characteristics associated with tumor molecular subtypes.^[^ ^214^ ^]^ They identified key mutations, such as KRAS, TP53, and SMAD4, validating that the organoids accurately reflected the genetic traits of tumors. WGS supports organoid modeling of tumor heterogeneity, clonal complexity, and mutation‐driven mechanisms. In 2023, organoids derived from 15 patients with high‐grade serous ovarian carcinoma (PDOs) exhibited distinct clonal populations with specific copy number variations (CNVs), which mimicked the intratumoral heterogeneity of patient tumors.^[^ ^215^ ^]^ However, the level of complexity varied, suggesting that different mutation processes might drive evolutionary changes in different ways.

Gene editing technologies, such as CRISPR/Cas9, are widely used in organoid functional research to explore the impact of specific gene mutations on tissue function or disease progression (Figure 4A). Ringel et al. constructed intestinal organoids and employed CRISPR screening technology to knock out specific genes.^[^ ^216^ ^]^ They then monitored gene expression changes within the organoids to verify their response to physiological functions, such as changes in the TGF‐β signaling pathway. Colon organoids derived from human colon tissue retained the molecular and phenotypic characteristics of colon tissue.^[^ ^217^ ^]^ By CRISPR‐editing specific genes (such as TGFBR2) and observing tumor formation and clonal dominance, they validated the ability of organoids to model patient‐specific gene mutations and their impact on tumor growth.^[^ ^217^ ^]^ Organoids constructed from the proximal small intestine of mouse embryos (FEnS) faithfully reproduced the process of intestinal development.^[^ ^218^ ^]^ CRISPR‐Cas9 knockout of key factors, such as Smarca4 and Smarcc1, promoted their transition from an immature to an adult‐like state, although their morphology remained spherical.^[^ ^218^ ^]^ The differentiation characteristics, however, became closer to adult intestine. Additionally, Liang et al. constructed mouse‐derived and human‐derived intrahepatic cholangiocyte organoids (ICOs), which could simulate the fate conversion between cholangiocytes and hepatocytes, closely resembling the characteristics of in vivo tissue.^[^ ^219^ ^]^ CRISPR screening confirmed that the organoids recapitulated the regulatory mechanisms of hepatocyte differentiation, with Fos and Ubr5 identified as key factors. Knockout of Fos promoted hepatocyte differentiation, accompanied by the upregulation of metabolic genes, indicating its role as a negative regulator. Knockout of Ubr5, on the other hand, led to differentiation defects and lipid metabolism abnormalities, suggesting its regulation of hepatocyte development through metabolic pathways.

The epigenome refers to all the epigenetic modifications that influence gene activity and expression without altering the DNA sequence.^[^ ^220^ ^]^ In organoid research, epigenomic evaluations help assess the characteristics and maturity of organoids, optimize culture conditions, and reveal developmental regulatory mechanisms (Figure 4A). These evaluations cover DNA methylation, histone modifications, chromatin accessibility, and non‐coding RNA regulation.^[^ ^221^ ^]^ In recent years, extensive research has focused on the epigenomic analysis of iPSC‐derived organoids (such as intestinal, brain, and retinal organoids) to uncover their epigenetic characteristics and regulatory mechanisms during development. Kraiczy et al. used iPSC‐derived intestinal epithelial organoids (iPSCo) to display typical intestinal characteristics, containing multiple intestinal cell types and expressing markers like E‐cadherin and LGR5.^[^ ^222^ ^]^ DNA methylation analysis showed that the methylation pattern of iPSCo was closer to that of the sigmoid colon (SC), although its epigenetic maturity had not yet reached in vivo levels. ATAC‐seq analysis of human hiPSC‐derived forebrain organoids revealed that chromatin accessibility patterns dynamically changed during developmental stages, progressively resembling fetal forebrain at mid‐ and late stages, particularly in regions associated with neurogenesis.^[^ ^223^ ^]^ hiPSC‐derived retinal organoids (hiPSC‐ROs) closely resembled the chromatin accessibility and developmental trajectory of in vivo retinal tissue, especially at early and mid‐stage key time points.^[^ ^224^, ^225^ ^]^ Histone modification analysis indicated that the active marker H3K4me3 co‐existed with the repressive marker H3K27me3, precisely regulating developmental genes.^[^ ^224^ ^]^ However, in the mature stage, the Notch signaling pathway activity was significantly lower than that in fetal tissue, leading to a reduced proportion of inhibitory neurons (such as horizontal cells and amacrine cells).^[^ ^225^ ^]^ In 2024, Zhang developed the scRCAT‐seq technique, which integrates ATAC‐seq and RNA splicing variant detection, allowing for synchronized analysis of chromatin accessibility and RNA variant expression in hiPSC‐ROs.^[^ ^226^ ^]^

#### 4.1.2 RNA

RNA‐based transcriptomics technologies, such as bulk RNA‐seq and scRNA‐seq (Figure 4A), analyze gene expression and transcriptional regulatory features to assess whether organoids reflect the functional state of the target tissue at the molecular level.^[^ ^227^ ^]^ RNA‐seq can reveal important gene mutations and expression patterns in organoid disease models, evaluating whether the activation of cancer‐related signaling pathways (e.g., Wnt, PI3K‐Akt) aligns with the primary tumor.^[^ ^228^, ^229^ ^]^ Wang et al. established organoids from tumor and normal colon tissues of colorectal cancer patients and performed scRNA‐seq.^[^ ^230^ ^]^ They found that tumor‐derived organoids replicated the gene expression features of cancer cells in vivo (e.g., Wnt signaling gene AXIN2), while normal colon tissue‐derived organoids exhibited tumor‐like features (e.g., Wnt signaling gene WNT6). In 2024, the Alcala team generated tumor organoids (PDTOs) derived from neuroendocrine tumor patients and used the Random Forest model to classify variations in RNA‐seq data, identifying key driver gene mutations (e.g., TP53, STK11).^[^ ^231^ ^]^ Through the analysis of gene expression patterns, cellular composition, and batch‐to‐batch variation, RNA‐seq technology not only helped validate organoid maturation markers and cellular differentiation potential, but also provided data support for organoid standardization and reproducibility. Phipson et al. guided iPSCs to differentiate into kidney organoids containing multiple kidney cell types and, through RNA‐seq, identified highly variable genes related to organoid maturation (e.g., NPHS1, PTPRO, NPHS2).^[^ ^232^ ^]^ Despite differences in cell type proportions between batches, the differentiation method for organoids demonstrated good reproducibility and robustness overall.

Spatial transcriptomics, through precise spatial localization and high‐resolution molecular analysis, not only uncovers complex developmental dynamics but also reveals intercellular relationships and molecular regulatory networks^[^ ^233^ ^]^ (Figure 4A). In 2022, a study combining spatial transcriptomics with single‐cell transcriptomics and long‐term 4D microscopy assessed the developmental dynamics of brain organoids derived from iPSCs.^[^ ^234^ ^]^ The results showed the heterogeneity and synchrony of neural progenitor cell (NPC) differentiation into neurons across different brain regions. In 2024, a novel spatial transcriptomics method, lamination‐based organoid spatially resolved transcriptomics (LOSRT), was developed for characterizing primary lung and liver organoids.^[^ ^235^ ^]^ LOSRT significantly enhanced the spatial resolution of primary organoid analysis. This method enables the analysis of organoid spatial structure at single‐cell resolution, identifying cell types and their spatial distributions, as well as revealing intercellular spatial relationships and potential interaction patterns. Additionally, spatial transcriptomics can be used to study the impact of external factors (e.g., light or drug stimulation) on organoid development. In 2023, spatial transcriptomics revealed that local activation of Sonic Hedgehog (SHH) signaling induced cell differentiation and spatial patterning in neural organoids, recreating typical structures similar to the dorsal‐ventral axis pattern observed during embryonic spinal cord development.^[^ ^236^ ^]^ In 2024, spatial transcriptomics (ST‐seq) was applied to identify the main cell types and their spatial distribution in ovarian organoids, and to assess the role of Tetrahydroxy Stilbene Glucoside (TSG) in promoting ovarian organoid development.^[^ ^237^ ^]^ The results showed that TSG significantly enhanced organoid development by regulating the interaction between vascular endothelial growth factor A (Vegfa) and EphB2 receptors, particularly boosting the proliferation and differentiation of oocytes and granulosa cells.

#### 4.1.3 Protein

Organoids should not only structurally resemble the target tissue but also maintain consistency at the molecular and functional levels, especially in terms of protein expression and function. Protein analysis, through the examination of protein expression, localization, modification, and activity in organoids, helps assess whether the organoids mimic the functional characteristics of the target tissue (Figure 4A). A proteomics study showed that gastric and intestinal organoids cultured in decellularized ECM exhibited cell differentiation and functional characteristics that were more similar to the native tissue.^[^ ^238^ ^]^ These organoids displayed higher levels of stem cell markers (e.g., Lgr5 in gastric organoids) and differentiation markers (e.g., Atp4b in gastric organoids, Lyz1 and Chga in intestinal organoids), with lower batch‐to‐batch variability and higher biocompatibility.^[^ ^238^ ^]^ High‐throughput protein detection techniques, such as isobaric tags for relative and absolute quantification mass spectrometry (iTRAQ‐MS)^[^ ^239^ ^]^ and sequential window acquisition of all theoretical mass spectrometry (SWATH‐MS),^[^ ^240^ ^]^ combined with real‐time monitoring, have been used to verify the authenticity of organoids in reproducing disease tissue protein expression, signaling, and functional characteristics. For instance, the construction of seven pancreatic organoids (four mouse organoids and three human organoids) covered stages from normal to invasive pancreatic cancer.^[^ ^239^ ^]^ iTRAQ mass spectrometry analysis revealed key molecular changes during pancreatic cancer progression, particularly in the regulation of metabolic and nuclear transport pathways. Some proteins (such as ACSM3, NT5E, GCNT3) showed similar changes in mouse organoids, which were also validated in human pancreatic cancer tissue. Another SWATH‐MS proteomics study indicated that colorectal cancer (CRC) patient‐derived organoids (PDOs) accurately reproduced the protein expression features of the patient's tumor, especially key signaling pathways like Wnt, MAPK, and PI3K.^[^ ^240^ ^]^ Furthermore, drug response experiments elucidated the resistance mechanisms of PDOs and revealed tumor heterogeneity through protein expression differences across various PDOs. Protein detection technologies also assist in evaluating neurodegenerative disease models, providing insights into molecular abnormalities in these diseases. Liu et al. compared normal mouse olfactory epithelial organoids (OE organoids) with OE organoids derived from Alzheimer's disease (AD).^[^ ^241^ ^]^ Olfactory dysfunction is an early symptom of AD and is associated with abnormal accumulation of β‐amyloid (Aβ) and Tau proteins. Immunofluorescence and ELISA assays revealed significant accumulation of Aβ and Tau proteins in the AD‐OE organoids at both early and late stages, with pathological severity worsening over time.

#### 4.1.4 Metabolites

Metabolomics is the study of the composition and changes in metabolites (such as small molecules) within an organism, offering multidimensional data to assess the functional status, physiological traits and pathological responses of organoids^[^ ^242^ ^]^ (Figure 4A). By comparing the metabolic profiles of organoids with native tissues, metabolomics ensures that organoids exhibit similar metabolic phenotypes. Metabolomic analysis of intraorganoid fluid (IOF) and extraorganoid fluid (EOF) secreted by endometrial epithelial organoids (EEOs) revealed significant metabolic differences between IOF and EOF.^[^ ^243^ ^]^ This asymmetry in secretion closely mirrors the secretion pattern of the in vivo endometrial epithelium, accurately reflecting the characteristics of the uterine fluid in the body. Through metabolomics, energy metabolism (e.g., glycolysis, oxidative phosphorylation) and metabolic activity (e.g., amino acid metabolism, lipid metabolism) can be assessed, providing insights into the organoid's functional state and identifying metabolic biomarkers associated with physiological or pathological conditions. Ilieva et al. constructed brain organoids derived from patients with autism spectrum disorder (ASD).^[^ ^244^ ^]^ Metabolomic studies revealed that in the early developmental stage (Day 39), the ASD organoids showed significantly enhanced glycolysis, which provides energy for cell proliferation. However, a reduction in oxidative phosphorylation and mitochondrial dysfunction might limit the brain's energy supply, impairing normal neuronal development. Metabolic data from malignant rhabdoid tumor (MRT) organoids indicated a more significant dependence on nucleotide synthesis compared to other tumor types or normal kidney organoids.^[^ ^245^ ^]^ Despite some metabolic heterogeneity among organoids from different patients, the reliance on nucleotide metabolism remained a common feature of MRT organoids.

### 4.2 Cellular Dimension

Cellular‐level evaluation is a key component in assessing the biological function and organizational characteristics of organoids. A comprehensive analysis, ranging from cellular functions and biological secretions to mechanical properties, can provide deep insights into the cellular activity, tissue microenvironment and the resemblance between organoids and the target tissue. This section will systematically explore evaluation methods from three aspects: cellular biological functions, cellular secretomes and cellular mechanical properties, providing important references for the study of organoid functionality and stability (Figure 4B).

#### 4.2.1 Cellular Biological Function

Cellular biological functions (such as cell viability, proliferation and senescence) directly impact the quality, stability and application potential of organoids (Figure 4B). Cell viability reflects the survival rate and physiological state of the cells within the organoid. High cell viability indicates that the organoid is suitable for further experimental research. Proliferative ability reflects whether the cells are in a state of rapid division and growth. For instance, 3D human dental pulp organoids exhibited a cell survival rate exceeding 95% on day 7, with vitality maintained above 90% for up to 4 weeks.^[^ ^246^ ^]^ ATP assays showed that the cells continued to divide from day 7 to day 21, but the proliferation rate was lower, reflecting the maturation characteristics of the tissue. Cellular senescence is another critical indicator for evaluating organoid performance. Senescent cells stop dividing and secrete senescence‐associated secretory phenotypes (SASP), which can affect the functionality of organoids and experimental outcomes. Yang et al. innovatively combined traditional biomarker detection with artificial intelligence to achieve efficient and accurate evaluation of cellular senescence in gastric organoids.^[^ ^247^ ^]^ Organoids derived from normal gastric mucosa showed senescence characteristics after a few passages, while some gastric cancer organoids could maintain proliferative ability for a longer period. Moreover, interdisciplinary technologies are also applicable for cellular‐level evaluation after organoid construction, revealing the functional state and developmental characteristics of specific cell layers within the organoid. For example, using liquid metal 3D microelectrodes to analyze retinal organoid development, specific cell layers such as retinal ganglion cells (RGCs) were precisely localized.^[^ ^248^ ^]^ This allowed for the recording of action potential waveforms, firing frequencies, and synchronized activities, revealing that their early developmental characteristics resembled those of the human fetal retina.

#### 4.2.2 Cell Secretomes

Cell secretomes include various cell membrane molecules, macromolecules and proteins, reflecting the physiological state of organoids, promoting cell‐to‐cell communication and responding to environmental changes (Figure 4B). A typical example is the interaction between endothelial cells and other cell types in vascularized organoids through secretomes. Yang et al. successfully generated heart organoids (vcCOs) with vascularization, chamber structures, and spontaneous beating capabilities.^[^ ^249^ ^]^ Ligand‐receptor pairs, such as COL4A2‐α2β1 and CADM1‐CADM1, between cardiomyocytes and endothelial cells, can activate the PI3K‐AKT pathway and Rho GTPase pathway, regulating endothelial cell migration, enhancing the anti‐apoptotic ability of cardiomyocytes, and maintaining the structural integrity of both myocardium and vasculature. In the tumor microenvironment (TME), interactions between tumor cells, immune cells, and stromal cells remain a major research focus in tumor organoid studies.^[^ ^250^ ^]^ For example, immune cells secrete cytokines and chemokines that influence tumor growth while performing immune surveillance functions.^[^ ^251^ ^]^ Tumor cells, on the other hand, secrete immunosuppressive factors to inhibit immune cell function, enabling immune escape.^[^ ^251^ ^]^ Stromal cells support tumor growth, invasion, and metastasis through the ECM, cytokines, and cell–cell contact.^[^ ^252^ ^]^ Patient‐Derived Lymphoma Organoids (PDLOs), containing native TME, demonstrate the complex interactions between various cells in the tumor microenvironment, especially the dynamic relationships between tumor cells and T cells, macrophages, dendritic cells, and B cells.^[^ ^253^ ^]^ These interactions successfully reproduce the immune escape mechanisms in follicular lymphoma and provide new strategies for optimizing immunotherapies, such as bispecific antibodies.

#### 4.2.3 Cellular Mechanical Properties

Cellular mechanical properties refer to the physical attributes of cells, such as stiffness, elasticity, adhesion, and deformability, which reflect the functional state, growth environment, and disease‐related changes of organoids^[^ ^254^ ^]^ (Figure 4B). The mechanical characteristics of different cell types reflect their cellular features and gene regulatory mechanisms, driving specific biological behaviors such as migration, invasion, and adaptation to the microenvironment. For example, a systematic analysis of the Young's modulus (E value) distribution on the basal surfaces of eight types of organoids using HS‐SICM technology showed that, compared to benign tumor cells and normal cells, malignant tumor cells have softer basal surfaces.^[^ ^255^ ^]^ This property supports their ability to traverse the matrix barrier and adapt to heterogeneous microenvironments, thereby enhancing their invasion and migration capabilities. Cellular mechanical properties play a crucial role in regulating biological functions such as tissue formation, morphology maintenance, and environmental adaptation. Polez et al. successfully quantified intercellular adhesion forces, such as cadherin‐mediated adhesion, using Atomic Force Microscopy (AFM), Single‐Cell Force Spectroscopy (SCFS) and revealed that intercellular adhesion is a key factor driving the formation of organoid spheroids.^[^ ^256^ ^]^ Using nanoindentation platforms and optical flow techniques to assess the mechanical properties of cells in 3D organoids, it was found that external forces significantly alter the structure of the cytoskeleton (e.g., actin filaments).^[^ ^257^ ^]^ These changes reflect mechanical properties and the cells’ ability to adapt to the environment through deformation, cytoskeletal remodeling, and displacement.

### 4.3 Organ Dimension

Organ‐level evaluation is an important dimension for assessing the overall structure and functional performance of organoids. Through morphological analysis, histological studies, and functional assessments, the characteristics of organoids in terms of 3D structure, cellular organization, and physiological functions can be systematically revealed. This section will explore key indicators for organoid evaluation at the organ level from three aspects: morphology, histology, and functional analysis (Figure 4C).

#### 4.3.1 Morphological Analysis

Tissue morphology determines the structure of the tissue, which in turn influences the temporal progression of cell fate (Figure 4C). Morphological changes reflect dynamic characteristics of the cytoskeleton, differentiation status, and signaling pathways. Morphological changes in breast cancer organoids can determine whether epithelial (smooth and round) to mesenchymal (spiky) transition, or its reversal, has occurred.^[^ ^258^ ^]^ Another study found that different preparation protocols (e.g., growth factors, exposure to Matrigel, and changes in orbital oscillation speed) significantly influenced the morphology of brain organoids.^[^ ^259^ ^]^ Organoids with more complex morphology showed transcriptional profiles closer to in vivo developmental states. The size and structure of organoids are closely related to their viability. Phenotypic data from colon cancer PDOs showed that organoid size was positively correlated with vitality.^[^ ^260^ ^]^ Additionally, training organoid morphological data using a Random Forest classifier (LDC, Live‐Dead Classifier) effectively predicted organoid survival status.^[^ ^260^ ^]^ Moreover, organoid morphology can be an important basis for disease diagnosis. Rectal organoid morphology analysis (ROMA) is used to diagnose cystic fibrosis (CF).^[^ ^261^ ^]^ By evaluating the morphological characteristics of organoids, it provides supplementary diagnostic information, with non‐CF organoids showing a regular morphology and a central lumen, while CF organoids exhibit irregular morphology and lack a central lumen.

#### 4.3.2 Histological Analysis

Histological analysis evaluates the differentiation status, spatial heterogeneity, and similarity to target tissues by observing the tissue structure, cell distribution, and specific markers in organoids (Figure 4C). It is an essential tool for validating the functionality of organoids and disease modeling. For example, in an Alzheimer's disease (AD) model, brain organoid slices spontaneously form amyloid plaque‐like and neurofibrillary tangle‐like (NFT‐like) structures in vitro, demonstrating the validity of the AD model.^[^ ^262^ ^]^ Cellular heterogeneity within organoids is a critical indicator of their functionality. By combining histological analysis with mass spectrometry imaging, the cell proliferation and spatial heterogeneity of lipid distribution in different regions of intestinal organoids can be assessed, indirectly proving that the organoids recapitulate certain features of the in vivo intestinal environment.^[^ ^263^ ^]^ Histological analysis is also crucial for verifying the authenticity of tumor organoids. Malignant peritoneal mesothelioma organoids (MPMOs) highly replicate the morphological features (cystic structure and dense tissue) and marker expressions (such as CK5/6, WT‐1) of the original tumor at the tissue level.^[^ ^264^ ^]^ Furthermore, advanced imaging techniques provide a more comprehensive display of the organoid's 3D structure. For instance, Stimulated Raman Histology (SRH) allows for dynamic monitoring and 3D virtual histological analysis while preserving organoid viability.^[^ ^265^ ^]^ It can also verify the high molecular and tissue‐level similarity between tumor organoids (tumoroids) and the original tumors.

#### 4.3.3 Functional Analysis

Organoid functional analysis (such as stability, mechanical properties, secretory functions, and metabolic levels) accurately reflects their physiological and pathological characteristics^[^ ^266^, ^267^ ^]^ (Figure 4C). This makes it a key tool for studying developmental mechanisms, modeling disease progression, screening drug efficacy, and exploring cell–environment interactions. Long‐term culture stability ensures the reliability of experimental data, improves the reproducibility of studies, and supports in‐depth research on complex diseases. For example, fallopian tube organoids can be cultured stably for over 16 months, maintaining tissue structure, cellular polarity, and functional stability throughout the experiment.^[^ ^38^ ^]^ Mechanical properties are an important parameter for evaluating organoid performance, reflecting their developmental and organizational maturity as well as their ability to adapt to external stimuli. For instance, the Young's modulus of brain organoids at different developmental stages is positively correlated with the development time, and external chemical stimuli (such as ethanol or blebbistatin) can either enhance or reduce the mechanical properties of the organoids.^[^ ^268^ ^]^ Organoids exhibit significant mechanical responses to physical, chemical, and biological stimuli. The use of a soft resistive force‐sensing diaphragm system enables dynamic monitoring of the mechanical properties of cardiac organoids under various stimuli.^[^ ^269^ ^]^ For example, electrical stimulation causes a rapid response, which gradually returns to baseline; carbachol treatment shows dose‐dependent changes and arrhythmias; and under diabetic conditions (high glucose and fatty acid environments), diastolic time is significantly prolonged, validating the effectiveness of the system in disease modeling.^[^ ^269^ ^]^

Long‐term morphological and functional tracking remains a critical challenge for organoid functional assessment. In recent years, non‐invasive optical imaging techniques, such as spectral‐domain optical coherence tomography (SD‐OCT), calcium imaging, and Fluorescence lifetime imaging microscopy (FLIM) combined with phasor (FLIM‐Phasor) analysis, have been employed to monitor the structural and functional dynamics of cardiac and intestinal organoids in real time.^[^ ^270^, ^271^ ^]^ Light‐sheet microscopy combined with genetically encoded fluorescent markers enables continuous observation of brain organoids across different developmental stages.^[^ ^272^ ^]^ In addition, flexible 3D microelectrode arrays (kiri‐electronic) allow electrophysiological recordings of brain organoids for up to 120 days under suspension culture conditions, while preserving their morphology and cellular composition.^[^ ^273^ ^]^ Together, these approaches provide a multi‐parameter, continuous, and quantitative platform for organoid functional tracking and supporting their applications.

### 4.4 In Vivo Dimension

The in vivo dimension of organoids refers to methods that place organoids within a living organism to promote maturation and assess their functions, including maturity, vascularization, integration, behavioral patterns, and the utility of disease models (Figure 4D). Whether organoids can establish effective vascularization and functional integration with host tissues is key to evaluating their maturity and practical use. For example, poly(lactide‐co‐glycolide) scaffold‐human lung organoid (HLO) complexes were transplanted into the epididymal fat pad of mice, forming airway‐like structures similar to adult lungs and exhibiting vascularization and cellular differentiation characteristics.^[^ ^274^ ^]^ The in vivo environment not only promotes further maturation of organoids but also provides a realistic background for validating their utility as disease models. A study showed that human brain organoids transplanted into the mouse brain exhibited neuronal differentiation, glial generation, and axon extension into multiple regions of the host brain.^[^ ^275^ ^]^ Building on this, Revah and colleagues utilized the cortical plasticity of neonatal immunodeficient rats and transplanted human cortical organoids into them.^[^ ^276^ ^]^ The transplanted organoids not only matured in vivo but also regulated host behavior at the neural circuit level. Additionally, this team established Timothy Syndrome (TS) patient‐derived iPSCs to generate cortical organoids and compared them to healthy organoids. While TS organoids did not show significant defects in vitro, transplantation into the in vivo environment revealed defects (dendritic morphological defects and increased synaptic density), highlighting the critical role of activity‐dependent signals in TS developmental defects. The in vivo environment provides fundamental support for constructing complex immunobiological systems in organoids. Human intestinal organoids transplanted into humanized immune system mice can mimic the development of intestinal immune tissue and immune responses under microbial exposure, demonstrating immune characteristics similar to those of human fetal intestines and enabling immune activation.^[^ ^56^ ^]^

## 5 Application of Organoids

**Figure 5:**
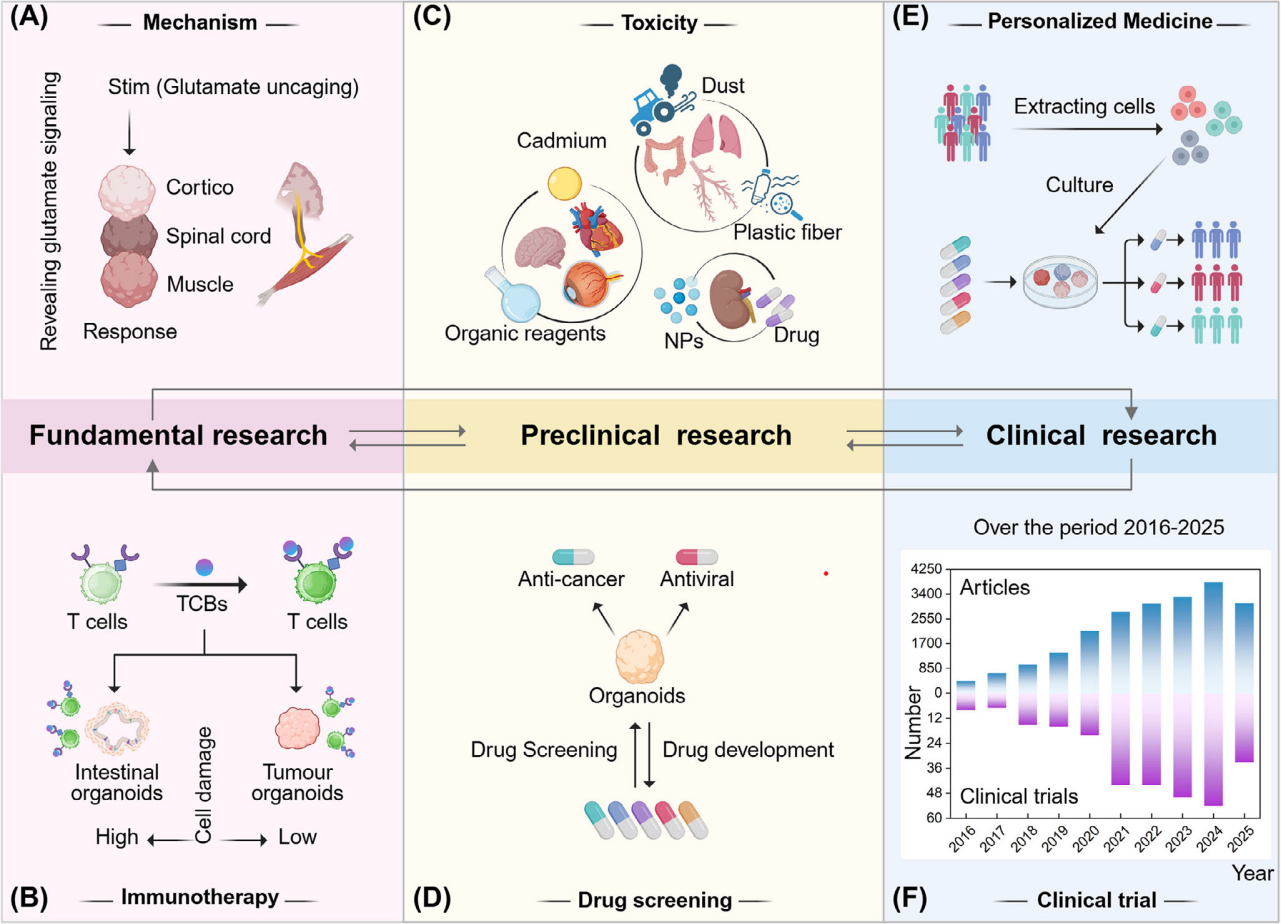
Three areas of organoids applications: basic research, preclinical studies, and clinical research.

Organoid technology is expanding its applications in the biomedical field, covering three major areas: basic research, preclinical studies, and clinical research (**Figure** **5**). In basic research, organoids are used to analyze disease mechanisms and develop novel immunotherapy strategies. In preclinical studies, organoids are applied to assess drug toxicity and screen candidate drugs. In clinical research, organoids are driving advancements in personalized medicine and clinical trials. This section will detail the key applications and developmental potential of organoid technology across these three levels.

### 5.1 Fundamental Research

This section discusses the applications of organoids in simulating neural signal transmission, the tumor microenvironment and infection mechanisms, analyzing how they reveal the occurrence and development patterns of diseases (Figure 5A). Additionally, it explores the role of organoids in immunotherapy, including drug efficacy evaluation and side effect studies, highlighting their significant value in both basic and translational medicine (Figure 5B).

#### 5.1.1 Mechanism

The study of organoid mechanisms is a key aspect of organoid applications, offering insights into the fundamental principles of organoid modeling of in vivo organ development, function, and pathological states. The mechanisms underlying organoids include gene regulation, secreted factor modulation, and cell‐to‐cell interactions. The occurrence of many diseases is linked to the abnormal activation or inhibition of cell signaling pathways, where key genes in these pathways play essential roles in signal transduction. Organoid research has revealed that various diseases (such as cortical malformations, neurodevelopmental disorders, and cholangiocarcinoma) are associated with specific gene mutations (e.g., CDK5RAP2, MECP2, CHD8) and abnormal gene pathways (such as the Fanconi anemia pathway), providing new insights into disease mechanisms, drug sensitivity, and resistance.^[^ ^277^, ^278^, ^279^, ^280^, ^281^, ^282^ ^]^ Organoid technology can be used to build complex physiological models, such as the cortico‐spinal‐muscular pathway, to help understand the mechanisms of neural signal transmission. For example, by fusing cortical organoids with spinal cord organoids and connecting them to skeletal muscle spheroids, the researchers successfully constructed cortico‐motor assembloids^[^ ^43^ ^]^ (Figure 5A). The study revealed that glutamate signals were transmitted through spinal neurons to the muscle, ultimately inducing muscle contraction, validating the central role of glutamate‐dependent synaptic transmission in neural signal regulation. In disease treatment (including anti‐infection and anti‐cancer), attention should be given not only to the target cells (e.g., virus^[^ ^283^ ^]^ and tumor cells) but also to the cell microenvironment (e.g., tumor microenvironment) and cell‐to‐cell interactions (e.g., the neurovascular unit). For instance, the PCCO model (a 3D model combining human cortical organoids with pericyte‐like cells) revealed the core mechanism of SARS‐CoV‐2 infection in the central nervous system (CNS): pericytes are the key nodes for viral infection and spread, while astrocytes contribute to viral transmission through inflammation and injury.^[^ ^284^ ^]^ The disruption of the neurovascular unit may be the main route for the virus to enter the CNS. Cancer‐associated fibroblasts (CAFs) play a crucial role in the tumor microenvironment by regulating tumor cell proliferation, survival, and drug resistance, significantly affecting chemotherapy outcomes.^[^ ^285^ ^]^ Co‐culture of pancreatic ductal adenocarcinoma‐derived PDOs with CAFs significantly impacted chemotherapy responses. CAFs regulated the proliferation and survival pathways of tumor cells, leading to higher drug resistance in tumor organoids (e.g., to gemcitabine). CellPhoneDB analysis revealed that interactions between tumor cells and CAFs secreted factors, such as HGF, might be key mechanisms in epithelial‐mesenchymal transition (EMT) and drug resistance.

#### 5.1.2 Immunotherapy

Organoids, as physiologically relevant models, are emerging as crucial tools in immunotherapy research. Utilizing organoids to simulate immunotherapy can help researchers comprehensively and accurately evaluate therapeutic efficacy and adverse effects while elucidating the mechanisms of tumor immune evasion. The differential responses of immunotherapy in healthy and tumor tissues may influence its efficacy and safety, particularly for novel immunotherapeutic agents such as Bispecific T‐cell Engager Antibodies (TCBs).^[^ ^286^ ^]^To assess the toxicity and efficacy of TCBs, patient‐derived intestinal organoids and tumoroids were co‐cultured with immune cells.^[^ ^286^ ^]^ Healthy organoids exhibited greater sensitivity to TCBs, characterized by significant T‐cell infiltration and apoptosis, whereas tumor organoids, due to their immunosuppressive microenvironment, demonstrated reduced apoptotic activity (Figure 5B). The potential immunotoxicity of TCBs in healthy tissues necessitates careful consideration during drug development. Moreover, the integration of epithelial organoids with autologous tissue‐resident memory T (TRM) cells led to the construction of human intestinal immune organoids (IIOs), providing a highly relevant in vitro platform for studying immunotherapy‐associated adverse effects, such as immune checkpoint inhibitor‐induced colitis.^[^ ^287^ ^]^ This model revealed the pivotal role of the TNF signaling axis in TRM cell migration and epithelial damage. Furthermore, it was found that ROCK inhibitors specifically suppress T‐cell motility and mitigate epithelial injury.^[^ ^287^ ^]^ These findings suggest that ROCK inhibitors hold potential therapeutic value in regulating immune responses by reducing excessive immune cell migration and inflammation. Additionally, the IIO model can be employed for screening and evaluating anti‐inflammatory drugs. In conclusion, immunotoxicity and immune targeting remain critical challenges in immunotherapy. Organoids provide essential support for the screening and optimization of immunotherapeutic agents, offering a powerful platform for drug development and mechanistic studies.

### 5.2 Preclinical Research

This section will explore the applications of organoids in drug toxicity assessment, efficacy prediction, and high‐throughput screening (Figure 5C,D). We hope to promote the application of organoids in future preclinical research (drug development, personalized diagnosis and treatment) through specific case studies.

#### 5.2.1 Toxicity

Organoids can simulate the responses of various human organs to harmful environmental factors during absorption, distribution, metabolism, and excretion. Therefore, organoids serve as the in vitro platform for evaluating the toxicity of environmental pollutants, chemicals, and drugs. Different types of organoids, such as respiratory, intestinal, cardiac, brain, and kidney organoids, can be specifically used to assess the impact of various harmful substances on specific organ systems (Figure 5C). For example, respiratory, lung, and intestinal organoids related to absorption can assess the toxicity of physical and chemical factors like microplastic fibers and road dust, more accurately simulating exposure conditions while demonstrating higher biological activity and cellular heterogeneity.^[^ ^288^, ^289^ ^]^ Cardiac, brain, placenta, and retinal organoids related to distribution can be used to study the toxicity of harmful factors such as cadmium, alcohol, organophosphate flame retardants, and polybrominated diphenyl ethers on the cardiovascular system, neurodevelopment, placental barriers, and retinal development.^[^ ^290^, ^291^, ^292^, ^293^ ^]^ Kidney organoids, associated with metabolism and excretion, can be used to study the toxicity mechanisms of nanomaterials (e.g., quantum dots) and drugs,^[^ ^294^ ^]^ showing comparable or even higher sensitivity, responsiveness, and accuracy than human primary cells.^[^ ^295^ ^]^ Additionally, organoids, by maintaining self‐renewal and differentiation, can simulate the effects of long‐term or chronic exposure on cells and tissues, helping identify chronic toxicity and cumulative damage. Their ability to be repeatedly cultured under the same conditions significantly improves the accuracy of toxicity assessments, providing strong support for environmental protection, drug safety, and the development of new materials.

#### 5.2.2 Drug Screening

Organoids have significant potential in drug screening. By using patient‐derived organoid models, it is possible to accurately assess the response of individual tumors or diseases to different drugs and treatment regimens, thus predicting the efficacy and drug resistance of specific therapies in patients (Figure 5D). In studies of ovarian cancer^[^ ^296^ ^]^ and head and neck cancer, organoids have demonstrated relevance to clinical responses, enabling the identification of the most effective anticancer drugs and targeted therapies. For example, organoids derived from ovarian cancer patients were used to screen 22 anticancer drugs, finding that Carboplatin and Belinostat were the most effective.^[^ ^297^ ^]^ Patient‐derived head and neck cancer organoids exhibited correlations between their response to radiation therapy (RT) and chemotherapy with clinical responses, particularly in patients receiving adjuvant RT.^[^ ^298^ ^]^ These models were also used to screen multiple targeted therapies, including PIK3CA inhibitors (such as alpelisib) and PRMT5 inhibitors.^[^ ^298^ ^]^ Organoids can also be used to develop novel nanodrugs and evaluate their toxicity and antitumor effects. Wang et al. utilized ovarian cancer patient‐derived organoid models to create nanoparticles, Abplatin (iv), by combining Pt (IV) prodrug with human serum albumin (HSA).^[^ ^299^ ^]^ This nanodrug not only achieved low toxicity and targeted delivery but also showed significant antitumor effects against platinum‐resistant tumor cells in ovarian cancer organoids. Additionally, organoids have demonstrated their potential in infection models and antiviral drug screening. Bannier et al. constructed conjunctival organoids, which could be infected by HSV‐1, hAdV8, and SARS‐CoV‐2.^[^ ^300^ ^]^ HSV‐1 infection was inhibited by acyclovir, while hAdV8 infection was sensitive to cidofovir, showcasing the potential of organoids in infection models and drug screening.^[^ ^301^ ^]^ Furthermore, transplanting organoids into immunodeficient mice in conjunctival injury models resulted in the formation of tissues with structure and function similar to human conjunctiva.^[^ ^301^ ^]^ These studies illustrate that organoids provide a reliable experimental platform for evaluating drug efficacy, optimizing treatment strategies, and exploring new drug candidates.

### 5.3 Clinical Research

In clinical research, organoid technology, serves as an important tool bridging basic research and clinical application. This section will explore the applications of organoids in personalized treatment plan selection (Figure 5E), clinical trial optimization (Figure 5F), and evaluation of novel therapies.

#### 5.3.1 Personalized Medicine

Personalized treatment is a medical approach tailored to an individual's genetic, environmental and lifestyle factors, aiming to enhance efficacy, reduce side effects and optimize treatment outcomes. The rapid responsiveness of organoids can predict a patient's sensitivity to certain drugs (e.g., chemotherapy, targeted drugs), helping avoid ineffective treatments or those with severe side effects, thereby promoting the personalization and precision of cancer therapy (Figure 5E). In colorectal cancer (CRC), PDOs can rapidly assess the drug sensitivity and resistance of individual tumors through in vitro drug testing, optimizing treatment plans.^[^ ^302^ ^]^ For example, a PDO demonstrated strong sensitivity to oxaliplatin, and the patient subsequently experienced significant efficacy after receiving the CAPOX regimen.^[^ ^302^ ^]^ Furthermore, vascularized organoid models (VOM) provide a precise tool to simulate the tumor microenvironment in gastric cancer research.^[^ ^303^ ^]^ VOM can be used for patient‐specific drug testing, effectively predicting clinical responses to VEGFR2‐targeted therapy, optimizing treatment strategies, and advancing precision personalized cancer treatment.^[^ ^303^ ^]^ The integration of AI with organoids offers a novel tool for predicting patient drug responses. Recent studies have demonstrated that the transfer learning model PharmaFormer, employing a unique three‐stage strategy, can accurately distinguish drug responders from non‐responders in patients with colorectal, bladder, and liver cancers.^[^ ^304^ ^]^ Compared with models relying solely on cell line data, the version fine‐tuned on PDOs exhibits significantly improved predictive accuracy due to their high biological fidelity. Organoids, as an emerging technology in regenerative medicine, go beyond simple tissue replacement, offering multi‐dimensional therapeutic functions. For instance, skin organoids not only reconstruct structural integrity but also have functions in regulating inflammation and promoting angiogenesis.^[^ ^305^ ^]^ Humanized skin organoids are generated from the patient's own cells to avoid immune rejection, and combined with 3D bioprinting technology, they can create custom repair solutions tailored to the specific shape and size of the patient's wound.^[^ ^306^, ^307^ ^]^ Additionally, these organoids significantly reduce the expression levels of inflammation‐related genes (such as the IL‐17 signaling pathway), which is important for individualized regulation of the patient's inflammatory response.

#### 5.3.2 Clinical Trial

Since the introduction of induced pluripotent stem cells (iPSCs) from mouse fibroblasts in 2006, organoids have emerged as a crucial model system in biomedical research (Figure 5F). They have been widely applied in disease modeling, drug screening, toxicity testing, gene therapy, and cancer treatment. As of September 7, 2025, a total of 30 295 articles on organoids have been indexed in the Web of Science, and 303 organoid‐related clinical trials have been registered on ClinicalTrials.gov, of which 17 have been completed. Among these clinical trials, 176 are observational studies, while 128 are interventional studies. The interventional trials include Early Phase 1 (5 studies), Phase I (12 studies), Phase II (42 studies), Phase III (8 studies), and Phase IV (0 studies). The clinical applications of organoids span various fields, including cancer treatment (e.g., NCT05577689, NCT05727020), drug screening (e.g., NCT05351983, NCT06459791), anti‐infective therapy (e.g., NCT04278326, NCT06139224), immunotherapy (e.g., NCT06084676, NCT05890781), and the integration of artificial intelligence with organoid research (e.g., NCT05927298, NCT06317610). Organoid‐related clinical trials have been conducted in multiple countries, covering major global medical research centers and leading pharmaceutical nations, including but not limited to the Netherlands, China, Canada, South Korea, France, the United States, the United Kingdom, India, and Switzerland. These countries have made substantial investments in organoid research, clinical trials, and commercialization, actively driving technological advancements and clinical applications in the field. As an emerging research tool, organoids are playing an increasingly significant role in medical research, drug development, and clinical therapy, particularly in cancer treatment, personalized drug screening, and gene therapy. With the continued expansion of clinical trials, the potential and application prospects of organoids will be further unveiled, solidifying their position as a transformative technology in biomedical science.

In addition to the above application scenarios, biofabrication and biomanufacturing strategies also play an important role in advancing organoid research. Automated bioprinting techniques include extrusion bioprinting, inkjet bioprinting, laser‐assisted bioprinting, stereolithography bioprinting, and digital light processing (DLP).^[^ ^308^, ^309^ ^]^ Among these, volumetric bioprinting enables large‐scale construction of organoids while maintaining high cell viability.^[^ ^310^ ^]^ Moreover, scalable suspension culture and microfluidic‐based systems are increasingly employed to address the limitations associated with manual handling and batch‐to‐batch variability,^[^ ^45^, ^149^ ^]^ thereby laying the foundation for the standardization and large‐scale application of organoids.

## 6 Challenges and Perspective

The materials used for organoid construction can be categorized according to different scales, allowing for a multi‐perspective optimization of their functionality and performance. This multi‐scale approach has driven the refined design of organoid materials, enabling the design of materials to meet the needs of different hierarchical levels, from overall structure to molecular function. However, materials for organoid construction still require further breakthroughs and optimization. Nanomaterials, with their high surface area and tunability, can optimize cellular behavior and the 3D microenvironment. However, their potential toxicity and biocompatibility issues (such as oxidative stress and excess reactive oxygen species) may affect the stability and functionality of organoids. Additionally, the functionalization of nanomaterials is a complex process that requires precise regulation to achieve ideal cellular behavior modulation. Micromaterials support tissue formation by simulating the physical properties of the ECM, but there are technical bottlenecks in the precise control of their size, shape, and surface characteristics. Although methods such as microfluidics and spray drying can improve precision, their diversity and complexity still fail to fully meet the requirements of different types of organoids. At the same time, mitochondria, as key organelles for cellular metabolism and energy supply, play a critical role in the activity and distribution optimization within organoids. This presents higher demands for enhancing the long‐term stability and functional complexity of organoids. Natural hydrogels are widely used due to their excellent biocompatibility and cell adhesion properties. However, batch‐to‐batch variations and the complex composition of biologically derived materials limit standardization and reproducibility. In contrast, synthetic hydrogels achieve controllable mechanical properties through chemical design but lack the biological activity of natural hydrogels, making it difficult to meet the physiological requirements of specific organoids. Scaffolds, designed with fine microstructures, support cell attachment and 3D growth. However, existing materials still face limitations in optimizing pore structure and mechanical properties, making it difficult to balance strength and flexibility simultaneously.

This article divides the evaluation criteria of organoids into four dimensions, analyzing their functions and biological characteristics at the molecular, cellular, organ, and in vivo levels. It comprehensively reveals their structure, function, and dynamic changes. The multi‐dimensional evaluation of organoids, through integrating genetic and metabolic activities, cellular behavior and microenvironment interactions, tissue function integration, and in vivo performance, significantly enhances the credibility and scientific rigor of the assessment. However, existing research tends to focus more on the short‐term functional performance of organoids, lacking systematic and comprehensive analysis of their stability and functional maintenance in long‐term cultures. The application of organoids has gradually deepened from basic research to preclinical and clinical research, demonstrating significant value in disease mechanism analysis, drug development, and personalized medicine. Nevertheless, the actual application from clinical research to personalized treatment still faces challenges such as standardized production and quality control, physiological relevance optimization, and ethical and regulatory issues. These challenges need to be addressed through technological innovation and interdisciplinary collaboration.

Furthermore, the integration of AI and organoid technology is driving a new wave of intelligent advancement in organoid research. AI, particularly through machine learning algorithms, is revolutionizing scientific research and development.^[^ ^311^, ^312^, ^313^, ^314^, ^315^, ^316^, ^317^, ^318^, ^319^, ^320^, ^321^, ^322^, ^323^, ^324^ ^]^ It offers powerful tools for the efficient construction, precise evaluation, and translational application of organoids.^[^ ^46^, ^317^ ^]^ The 2024 Nobel Prize in Physics was awarded to John J. Hopfield and Geoffrey E. Hinton for their pioneering contributions to artificial neural networks and machine learning, underscoring the critical role of AI in interdisciplinary scientific innovation.^[^ ^325^ ^]^ In terms of organoid construction, AI can analyze large‐scale experimental datasets to optimize hydrogel matrix design, identify spatial architecture, fine‐tune culture conditions, and modulate external stimuli. These capabilities contribute to the generation of more physiologically relevant and functionally mature organoids. Moreover, AI enables rapid screening of construction strategies and intelligent control of bioreactor conditions, significantly improving the reproducibility and scalability of organoid production. For the assessment of organoids, AI‐powered image analysis overcomes the limitations of conventional manual methods, which are often time‐consuming and subject to human bias. Trained on large image datasets, AI algorithms can accurately extract key cellular features, such as cell count, morphology and behavioral dynamics as well as organoid‐level characteristics, including size, shape, internal structure and marker expression.^[^ ^326^, ^327^ ^]^ This facilitates high‐throughput, quantitative, and objective evaluation of organoid quality. Additionally, AI‐driven closed‐loop systems that respond to real‐time electrophysiological signals enable dynamic modulation and functional optimization of organoids.^[^ ^328^ ^]^ In terms of application, AI can integrate multi‐omics data derived from organoids to elucidate disease mechanisms and drug response pathways. For instance, AI‐assisted analysis of lung organoids infected with SARS‐CoV‐2 has revealed critical signaling pathways and identified potential therapeutic targets, supporting drug repurposing and novel target discovery.^[^ ^329^ ^]^ Furthermore, AI can predict drug responses and assist in personalized treatment planning.^[^ ^312^ ^]^ When combined with PDOs, AI enables individualized drug screening and “one patient, one model” approaches, greatly enhancing the precision of therapeutic interventions.^[^ ^330^ ^]^

In conclusion, this article, from a multi‐scale perspective, systematically explores the construction, evaluation, and application of organoids, providing a theoretical foundation and practical direction for optimizing organoid functions, improving the evaluation system and accelerating clinical translation, and addressing the role of AI for its development.

## Conflict of Interest

The authors declare no conflict of interest.
